# Patterns of human and bots behaviour on Twitter conversations about sustainability

**DOI:** 10.1038/s41598-024-52471-z

**Published:** 2024-02-08

**Authors:** Mary Luz Mouronte-López, Javier Gómez Sánchez-Seco, Rosa M. Benito

**Affiliations:** 1 Higher Polytechnic School, Universidad Francisco de Vitoria, Carretera Pozuelo a, Av de Majadahonda, Km 1.800, 28223 Madrid, Spain; 2 Grupo de Sistemas Complejos, Escuela Técnica Superior de Ingeniería Agronómica, Alimentaria y de Biosistemas, Universidad Politécnica de Madrid, Avda. Puerta de Hierro 2-4, 28040 Madrid, Spain

**Keywords:** Complex networks, Scientific data

## Abstract

Sustainability is an issue of worldwide concern. Twitter is one of the most popular social networks, which makes it particularly interesting for exploring opinions and characteristics related to issues of social preoccupation. This paper aims to gain a better understanding of the activity related to sustainability that takes place on twitter. In addition to building a mathematical model to identify account typologies (bot and human users), different behavioural patterns were detected using clustering analysis mainly in the mechanisms of posting tweets and retweets). The model took as explanatory variables, certain characteristics of the user’s profile and her/his activity. A lexicon-based sentiment analysis in the period from 2006 to 2022 was also carried out in conjunction with a keyword study based on centrality metrics. We found that, in both bot and human users, messages showed mostly a positive sentiment. Bots had a higher percentage of neutral messages than human users. With respect to the used keywords certain commonalities but also slight differences between humans and bots were identified.

## Introduction

According to the United Nations, sustainability refers to what makes it possible to meet current human needs without compromising the ability of future generations to meet their own necessities^1^. Sustainability is an issue of international concern. As a consequence, in 2015, world leaders adopted a set of 17 global goals (these objectives were: *SDG*1: No poverty, *SDG*2: zero hunger, *SDG*3: good health and well-being, *SDG*4: quality education, *SDG*5: gender equality, *SDG*6: clear water and sanitation, *SDG*7: affordable and clean energy, *SDG*8: decent work and economic growth, *SDG*9: industry, innovation and infrastructure, *SDG*10: reduced inequalities, *SDG*11: sustainable cities and communities, *SDG*12: responsible consumption and production, *SDG*13: climate action, *SDG*14: life below water, *SDG*15: life on land, *SDG*16: peace, justice, and strong institutions, *SDG*17: partnerships for the goals) to eradicate poverty, protect our planet and ensure prosperity, constituting the 2030 agenda for sustainable development^2^. Pieces of research exist that describe some approaches to achieving environmental protection, the so-called “triple-de”: decarbonisation, detoxification, and dematerialisation^3^. In addition to empirically analysing data for a number of companies over certain time periods referring to the companies’ environmental responsibility, there is also an examination of how media attention impacts the relationship between environmental protection and sustainable development^4–6^.

Social networks, due to their large number of users, are powerful tools to understand public perception. In particular, the online social site Twitter had 229 million of monetized daily active users in 2022 (Monetized Daily Active Users (mDAU) symbolises the number of unique users who access and interact in a given day)^7^. Twitter is characterised by the fact that the majority of its user accounts are public (Twitter offers its users two main privacy settings: public and private. In the case of private accounts, only those individuals the user follows are able to read their messages. It is important to note that Twitter does not allow different privacy settings for individual messages.), in turn, makes their messages public. By contrast, other social networks (for example Facebook have mostly private accounts, which makes their content only accessible to each user’s network of friends. In particular, Facebook allows users to choose from a variety of privacy options, allowing them to have a fully visible profile, a profile only viewable by recognised friends, or anything in between. Users can modify the privacy settings for each specific post, making it public, visible only to friends, private, or it can be set to a custom audience. The above is an intrinsic feature of both social networks, that have different approaches^8^.

For researchers Twitter has the advantage that it provides a powerful Application Programming Interface (API) which enables access to it in advanced ways^9,10^ and^11^. Using these API, it is possible to obtain a large amount of data concerning each tweet, user profile, place in which a tweet was sent, among other supplemental information^9,10^, and^11^. Since September 2017, Twitter users have been able to post messages of up to 280 characters each (previously it was limited to 140). Twitter users can publish new tweets, reply, retweet and quote^9^. Furthermore, it must be noted that, in other networks such as Facebook, which are more private in nature, accessing the information provided by its API is more complex^9,11^. In particular, on Facebook, retrieving many status messages is more complicated than the message retrieval that is possible on Twitter. Consequently, Twitter’s API provides greater potential to access all the information about a topic or discussion^9–11^ and^12^.

With regards to sustainability discourse on Twitter, certain research analysed the practice of tweeting about Corporate Social Responsibility and Sustainability issues, focusing on those which are the most relevant topics, and also considering who is leading the debates on these issues^13–15^. Previous research also explores how climate leadership and environmental messages impact on companies’ stock prices^16^. There are studies that place the main examination focus on companies within a particular country^17^.

Multiple approaches have been used to analyse social networks in depth and with the purpose of understanding their mechanisms of operation^18–20^, as well as to examine public perception on various topics of social interest^21–24^, including political electoral campaigns^24,25^, and polarisation issues^26–28^. Certain mechanisms governing the propagation of information from bots, and their influence on public opinion formation, have also been explored^29–36^. There are also several studies that have analysed the behaviour of bots on Twitter by examining temporal patterns that describe their activity^37–39^, such as entropy, existence of motifs (repetitive sub-sequences in the interaction time series), unusual periods of inactivity (discords) and detection of periodicities in the posting of messages.

With respect to machine learning models, including Random Forest, Generalised Linear, Support Vector Machine, and neural networks models, particularly, those using deep learning methodologies such as transformer-based models, have been widely used in various fields, such as: medicine^40–42^, economics^43–45^, environment^10,46^, industry^47–50^, and food security^51^, among others.

Taking into consideration all of the above, we aim to answer the following research questions:

1. What is the optimal model for identifying account typologies? Can existing models be improved?
2. Regarding sustainability, are there differences in the behavioural patterns on Twitter of humans and bots?
3. Are there various patterns of activity? What are their characteristics?
4. What is the sentiment on sustainability? Are there differences between humans and bots? How has the sentiment evolved over time?
5. What are the most relevant words used in the tweet text? Are there differences between humans and bots?

In this paper, we present an analysis of the messages posted on Twitter about sustainability with the goal to further understand social opinion on this issue. In particular, the said analysis can accurately identify the account typologies (bots or humans) that at some time made posts related to sustainability. Furthermore, useful information can be collected from knowing interaction patterns and understanding characteristics of these tweets. Specifically, the novelties of this research are: (i) The large database on which the study is built allows us to better understand the patterns of messages published by both humans and bots dealing with a specific topic (in particular sustainability). Notice that previous studies, as the one mentioned above used a much smaller data set. (ii) Building a model based on the digital footprint of each user, which symbolises all types of messages that a user sends, in a similar way as previosly proposed^34^. But in addition to that, we go one step further utilising various compression algorithms (gzip, zlib, bzip2, lzma and smaz) and comparing them. Analogously to^52^, the model is built using attributes related to the user’s profile and their activity (which is described through parameters associated with the messages sent by each author (see Sect. "Activity patterns"). At the same time, as a novelty, our model utilises these parameters together with others referring to the user’s digital footprint. Consequently, a robust model is developed providing optimal results. We apply our model to Twitter data corresponding to users who at some point during the period from 2016 to 2022 sent messages (tweets, retweets, quotes, or replies) related to sustainability. (iii) On the basis of the time series describing the activity of each user, behavioural patterns are detected using clustering analysis and statistical tests. As a novelty with respect to previous research^37–39^, the behaviour of humans and bots is studied based on parameters that globally characterise the time series corresponding to tweets, retweets, replies and quotes (see Sect. "Activity patterns"). (iv) We determine the most relevant sentiments and words, in addition to detecting some differences according to the activity pattern, the type of account, as well as the polarity of the messages.

## Results

### Compression algorithms

In this section, based on Twitter data downloaded for the period 2006-2022, we present the main results obtained in our study. The tweets used are those containing the following keywords: sustainable agriculture, sustainable food, renewable energy, green urban, sustainable transport, pollution, sustainable city, and sustainable industry (see Sect. "Building the dataset"). We used 38,615 users and 96,252,871 tweets (40,000 users were processed, but only those users with a public account were taken into account in the analysis, which were 38,615). The users for the analysis were randomly selected among all those who at some time sent a tweet with any of the above mentioned keywords related to sustainability. Then, a digital footprint was built for each user (see Sect. "Building the dataset"). It consists in a string of characters that represent the different interaction mechanisms in Twitter: post a tweet (’A’), retweet (’T’), reply (’C’) and quote (’G’) used by the user. As an example, a user’s footprint could be a string in the form “ACTCATTTTAG”, meaning that the user has made the following actions: post a tweet, reply, retweet, reply, post a tweet, 4 retweets, post a tweet and quote.

After creating the users’ digital footprint a compression algorithm was applied. We applied different compression methods in order to find the most appropiate one to our study. The compression methods used were: (gzip, zlib, bzip2, lzma and smaz). We found that gzip algorithm was the most optimal, see Sect. "Building the model").

**Figure 1:**
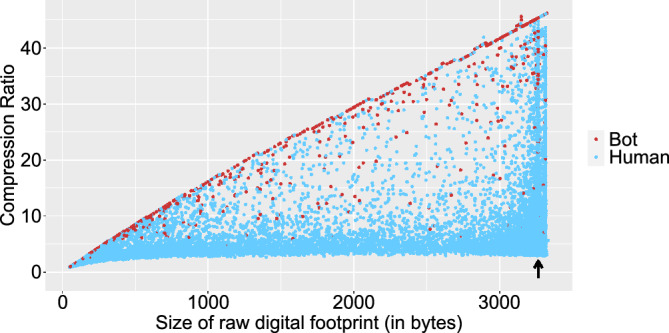
Compression ratio as a function of the size of the raw footprint for gzip algorithm. Compression ratio is calculated as: size of raw digital footprint (in bytes)/size of compressed digital footprint (in bytes). The arrow points to the critical point of 3265.

Figure 1, displays the compression ratio (size of raw digital footprint (in bytes)/size of compressed digital footprint (in bytes)) as a function of the size of the raw footprint for bot and human users (in bytes), both magnitudes are highly relevant^34^ for gzip method. It can be seen that this ratio showed a gap between human (dots in blue colour) and bot (dots in red colour) users. For human users with footprints smaller than 3,265 bytes, the compression ratio was in most cases less than 10. However, if the sizes were larger than 3,265 bytes, the ratio showed a large variability. This is in line with the fact that information with high entropy cannot be compressed/decompressed with optimal efficiency^53^. For bots with footprint sizes lower than 3,265 the compression ratio showed linear growth with size. The linear fit in addition to the raw footprint size that resulted in the highest variability of the compression ratio has been included in the Supplementary Material Document (Tables S1 and S2). It can be observed that bots exhibited a higher median of the raw footprint (see statistical quartiles in Table S3) than human users.

### Building the model

We built a supervised learning model based in the use of labelled data sets with the purpose of classifying our used Twitter data to discern whether a user is a bot or not. With the aim of labelling the users as humans or bots, the Botometer tool was utilised (see "Methods" Section). Botometer is a very appropriate resource to identify bots on social networks such as Twitter. However, because bot detection is a complex technique, it has limitations: the accuracy of identification is limited by the quality of the data, and the algorithms used for the analysis. Botometer provides a probability score that a user account is a bot, which can result in false positives and false negatives in detection. As bots become more sophisticated, they more optimally imitate human behaviour. This fact makes it difficult for bot detection tools to keep pace with the changing strategies of malicious users. Human user accounts also exist that tend to be identified as bots. This is because they exhibit repetitive behaviours, or utilise tools to automate certain tasks^54,55^. There is also a linguistic and cultural bias in bot detection algorithms, which implies that tools are not equally effective in all languages^55^. In order to mitigate the limitations of Botometer, we have carried out the following actions: (i) select only tweets in English, (ii) choose only Twitter users who have a public account and (iii) adjust the threshold value for labelling users as bot or non-bot in order to match the proportion of bots values that are indicated in other studies as typical on Twitter.

With respect to the model, it was built using a variety of approaches that correspond to the following three alternatives:

- Alternative 1: in this alternative, as input variables, we consider both raw size and compression ratio of the user’s fingerprint, similarly to^34^, but additionally we include several compression methods.
- Alternative 2: in this alternative, analogously to^52^, certain parameters referring to the messages from an author, as well as to the user profile were considered as input attributes. They were: ratio between amount of persons being followed and number of followers, percentage of retweets, replies, quotes and tweets over total messages. Also included, were the ratio of tweets with multimedia content, and maximum time gap between messages (in hours). No parameters related to compression algorithms were used
- Alternative 3: in this option, both alternatives 1 and 2 were taken together.

Various mathematical models were implemented (see Sect. "Building the model"), which were generalised linear (*GLM*), random forest (*RFM*) and support vector machine (*SVM*). They were evaluated utilising several performance metrics. The most optimal mathematical model and compression method were random forest and gzip, respectively. Although, lzma, bz2, zlib also showed a good accuracy (higher than 0.80). For each option of explanatory variables and mathematical model, the best hyperparameters (where they existed) and performance metrics, have been given in the Supplementary Material Document (see Tables S4–S17). The main performance metrics for the three aforementioned alternatives, gzip compression algorithm, and *GLM*, *RFM* and *SVM* procedures are given in Table 1.

**Table 1:** Performance metrics of the validation set for the models: generalised linear (*GLM*), random forest (*RFM*) and support vector machine (*SVM*) models, for the three alternatives considered.

	Alternative 1			Alternative 2			Alternative 3		
Metric	GLM	RFM	SVM	GLM	RFM	SVM	GLM	RFM	SVM
Accuracy	0.8334	0.8876	0.8111	0.8381	0.9000	0.8375	0.8015	0.8948	0.7974
Sensitivity	0.8206	0.8848	0.8206	0.8257	0.8966	0.8252	0.7863	0.8899	0.7804
Specificity	0.9373	0.9145	0.9393	0.9615	0.9331	0.9568	0.9519	0.9429	0.9624
Kappa	0.5864	0.5477	0.3993	0.4470	0.5839	0.4485	0.3834	0.5724	0.3826

gzip algorithm has been utilised in Alternative 1 and Alternative 3.

### Activity patterns

With the purpose to unveil the activity patterns of both humans and bots, a clustering analysis using the $K-Means$ method was implemented for A (tweet), T (retweet), C (reply) and G (quote) interactions. For each user, the following attributes were taken into consideration: mean, standard deviation, median, mode as well as maximum, and minimum number of tweets posted daily, including maximum used lag order and obtained *p*-*value* in the Augmented Dickey-Fuller (ADF) test^56–58^ (see Sect. "Activity patterns").

For the different types of messages analysed, 100 experiments were performed in order to study the interactions of human users. A number of human users identical to the number of bots were randomly selected with replacements in each trial. The clustering tendency was evaluated using the Hopkins statistician (*HS*). Silhouette and Dunn indexes were also computed to obtain the optimal number of clusters (see Supplementary Material Document, Sect. S2.3).

The results obtained for the different types of interactions are described below.

#### Type A interactions

Data from 3421 bots and 34,229 humans was used in the examination of type A messages. Based on the value provided by both Silhouette and Dunn indexes, the human users could be grouped into two clusters. The average value for *HS* was 0.01060, with a standard deviation equal to $1.1540 \times {10^{-05}}$ showing evidence of the existence of clusters (the closer this statistic is to 0, the more evidence in favour of the existence of clusters in the information exist.). The average values corresponding to Silhouette and Dunn indexes were 0.89673 and 0.02408. Taking the 100 experiments into account, the Kruskal-Wallis test^59^ showed that there was no difference between groups marked as 1 (group 1), nor between groups marked as 2 (group 2). The average percentage of human users in groups 1 and 2 was 2.24 % and 97.76 % respectively. Regarding bots, according to the Silhouette and Dunn indexes, two clusters were also detected, each having 8.39% and 91.61% of bots.

For human users, Table 2 displays the values of the median of the parameters exhibited by the centroids of each cluster in the 100 experiments performed. Table 2 shows magnitudes corresponding to centroids in each cluster for both humans and bots.

#### Type C interactions

In order to study the type C messages, 25,264 users (159 bots, and 25,105 humans) were analysed. A variety of the trials executed returned a different optimum number of clusters in accordance with the values of Silhouette and Dunn indexes. Because of this, we were not able to perform an activity pattern analysis.

#### Type T interactions

In relation to the type T messages, 202 bots and 28,866 humans were explored. The *HS*, calculated over the 100 experiments, provided an average value equal to 0.06259 with a standard deviation equal to 0.00160. According to the Silhouette and Dunn indexes, 2 was the optimum number of clusters in all trials. On average, each cluster included 7.83% and 92.17% of the total human users. Regarding bots, 3 clusters containing 14%, 4%, and 82 % of the total users were found.

For human users, Table 2, shows the median of the parameters associated with the centroids of each cluster in the 100 experiments. For bot users, the attributes corresponding to the centroids are displayed in each cluster.

#### Type G interactions

For Type G messages, 3,626 bot and 34,970 human users were examined. Notice that although there is a considerable higher number of bots than in the other types of interactions, the analysis did not detect any cluster either for humans or bots. For human users, the *HS* showed average and standard deviation values equal to 0.15265 and 0.0011. For bots this statistic was 0.13584.

**Table 2:** Results of the clustering analysis for the Types of interactions A (post tweets) and T (retweets).

AT	C	*lag*	*p*-*value*	*mean*	*sd*	*mode*	*median*	*max*	*min*
Type A messages									
B	1	2.60627	0.40886	143.98569	71.93038	6.25784	143.47213	293.790941	31.90244
	2	6.23772	0.14429	15.46559	7.60016	6.32419	14.87795	40.40587	2.97064
H	1	3.52000	0.21480	101.14975	63.35272	5.04000	96.14000	281.98667	20.24000
	2	6.64663	0.05713	4.77276	3.22845	2.26640	4.02640	22.75923	1.26548
Type T messages									
B	1	2.85714	0.23382	71.91453	61.53571	48.16915	6.28571	195.39286	8.96429
	2	2	0.50036	204.21422	206.50000	95.08581	4.22222	359.77778	43.22222
	3	2.96970	0.28107	2.48518	1.78788	1.910903	1.29091	9.77576	1.01212
H	1	3.61538	0.17658	31.00415	23.46937	7.69231	26.38461	123.46154	3.38462
	2	5.16340	0.09353	2.60786	2.24073	.29412	1.81699	16.53595	1.02614

For human users, the values of the parameters shown in the table correspond to the median calculated over the 100 experiments. The meaning of the abbreviations is: *lag*: used lag order in ADF test. *H*: humans, *B*: bots, *p*-*value*: obtained *p*-*value* in the ADF test. *mean*, *sd*, *median*, *max* and *min* represent the mean, standard deviation, median, maximum and minimum number of messages.* AT* account typology,* C* cluster.

### Account typologies

This section aims to investigate whether differences exist between humans and bots behaviour in tweetting. To do this, we analyze the values of different statistical such as the mean, standard deviation, median, mode, maximum, and minimum number of tweets sent daily, in addition to used lag order and obtained *p*-*value* in the ADF test. For A and T messages, the Kruskal-Wallis test demonstrated that considering the above-mentioned factors individually, there were no dissimilarities between the 100 trials performed (*p*-$value>0.05$). Therefore, it was possible, without loss of generality, to utilise a single experiment to examine differences with the bot group.

For Type A messages, for all considered parameters except the median, a *p*-$value > 0.05$ was obtained in the Kruskal-Wallis test proving that a differentiation exists for this magnitude. In relation to Type C, G and T messages a *p*-$value>0.05$ was obtained for all parameters individually analysed.

### Sentiment analysis

With the purpose of examining the sentiment of the interactions on sustainability, similarly to^9,11^, only type A messages were taken into consideration, because they are the only ones that bring new personal opinions.

The sentiment from the original tweets posted during the period 2006-2022 was analysed. According to the procedure indicated in Sect. "Building the dataset", 40,000 users were selected, but due to the fact that some of them had an empty author field, only 37,650 were considered. For bots and humans, the average text size of each tweet was 114 and 130 characters, respectively.

In the same way to^9,11^ for each tweet the sentiment is calculated as an average of the sentiment of the words included in it. The computed sentiment is typified as positive (when its value is in the interval [0, −1]), neutral (if it is 0) and negative (when its value is in the interval [−1, −0]). Only tweets with a subjectivity higher than 0 were considered.

We observed that bot users showed a higher number of neutral tweets than human users. For each user typology and sentiment type (positive, negative and neutral), we built a 17-dimensional vector $v_{tucs}$, in which each component is the annual average sentiment. For each user typology and period analysed, we create three vectors, describing the positive, negative and neutral sentiments.

Sentiments by user typology, *ut*, humans or bots:

$${\text{positive}}: \,\vec{v_{{utpos}}} = (v_{{utpos1}} ,v_{{utpos2}} ,...,v_{{utpos16}} ,v_{{utpos17}} )$$

$${\text{negative: }}\vec{v_{{utneg}}} = (v_{utneg1}, v_{utneg2},..., v_{utneg16}, v_{utneg17})$$

$${\text{neutral: }} \vec{v_{{utneu}}} = (v_{utneu1}, v_{utneu2},..., v_{utneu16}, v_{utneu17})$$

In order to unveil differences between human and bot users behaviour, the cosine similarity between vector pairs corresponding to each sentiment type was calculated. This metric, which presents values in the range [−1, 1], is defined as follows^60^:

1 $$\begin{aligned} Cosine \, Similarity \ (a,b) =\frac{\sum _{ij}^Na_ib_j}{\sqrt{\sum _{ij}^N a_ia_j}\sqrt{\sum _{ij}^N b_ib_j}} \end{aligned}$$

where *a*, *b* symbolise two N-dimensional vectors. $a_{i}$ and $b_j$ represent the coordinates of each vector. *N* is the dimension of each vector.

When the angle between the two vectors (a and b) is small it generates high cosine values, (close to 1) indicating high cosine similarity.

**Figure 2:**
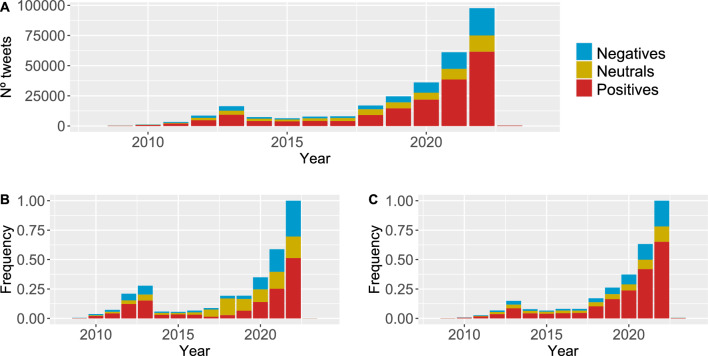
Evolution of sentiment polarity. (**A**) all users, (**B**): Bots, (**C**) Humans got from tweets posted by 37,650 users about sustainability.

It was observed that, despite the relevant differences in the number of tweets between user typologies (during the analysed period, a maximum 80,000 messages were posted by humans in a year and 14,000 by bots), the cosine similarity was 0.95 for negative and positive sentiment vectors. Neutral sentiment showed a higher difference between both user typologies with a value equal to 0.84. Figure 2 depicts the historical evolution of the sentiment about sustainability (utilising the chosen keywords for the tweets downloaded).

**Figure 3:**
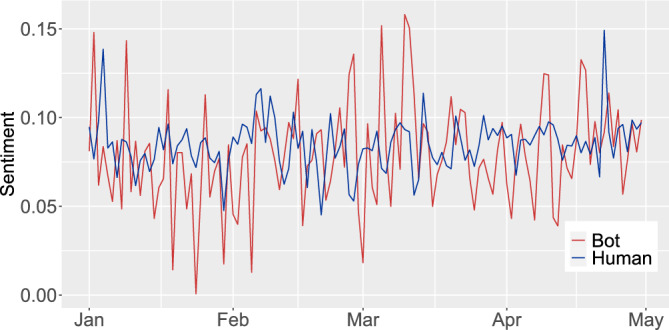
Evolution of the sentiment about sustainability got from tweets posted by 37,650 users during the first 4 months of 2022.

Figure 3 shows the evolution of the average daily sentiment during the first four months of 2022 for both human and bot users. It can be seen that the messages posted about sustainability by bots exhibited a higher range of variability in the daily average sentiment. It varied between a value of 0.001, which is very close to neutrality, up to a value of 0.158. In contrast, the daily average sentiment of human users varied between 0.045 and 0.149. The bots also presented more messages whose average sentiment was located in the aforementioned extreme values (see Supplementary Material Document, Table S18).

### Keyword analysis

For each tweet, we obtained the most frequent words, as well as the most relevant ones according to centrality metrics^61^ (see Sect. "Keywords analysis"). The latter could be assimilated to the process that a human being performs when visualising a text and deduce the most significant content.

For each user typology and cluster, Table 3 shows the top 20 keywords. It can be observed that there are 5 words that are common in all clusters. They are “air pollution” and “new renewable energy” which symbolise important topics in sustainability matters. There are also other common words between clusters, if the results derived from calculating the centrality metrics are compared with those obtained by applying the counting method. It can be seen that some low frequency words are, however, a key element to understand the content of the tweet (see Table 3 and in Supplementary Material Document, Table S20).

**Table 3:** Top keywords related to sustainability got from tweets posted by 37.650 users by cluster and typology (TKG method).

All	Bots cluster 1	Bots cluster 2	Humans cluster 1	Humans cluster 2
**air**	**air**	**air**	**air**	**air**
city	bike	**energy**	city	city
**energy**	Delhi	facebook	**energy**	clean
facebook	**energy**	green	food	**energy**
food	new	new	green	food
new	**pollution**	**pollution**	industry	green
**pollution**	**renewable**	power	new	industry
power	tweet	project	**pollution**	new
project	ecoops	quality	power	**pollution**
**renewable**	sustainable	**renewable**	project	power
solar	company	solar	**renewable**	project
sustainable	green	sustainable	solar	**renewable**
via	instead	water	sustainable	solar
water	around	micron	twitter	sustainable
green	due	consider	agriculture	twitter
twitter	emission	current	clean	water
industry	food	good	water	make
wind	good	index	climate	climate
clean	motor	moderate	facebook	facebook
future	place	particle	learn	health

Common words to all the clusters are marked in bold.

## Discussion

This research makes a relevant contribution to its application area by extending the work of^34^ and^52^, implementing a model that distinguishes between human and bot accounts on Twitter. The incorporation as explanatory variables of the parameters related to footprint compression, profile characteristics and user activity, improves the model’s performance. The model was tested with five different compression algorithms (zlib, bzip2, lzma and smaz), showing good results (accuracy $>0.80$) with four of them slightly favouring gzip as the most optimal. The sample taken into consideration for the construction of the model consisted of 38,615 users with the percentage of bots ranging from 9 to 15 percent^62^.

The time series characterisation associated with each user typology allowed us to perform a clustering analysis for both type A and T messages. For bot and human users, the analysis of type A messages demonstrated the existence of two clusters, pointing out that there were two different ways of operating on Twitter. For bot and human users, type T messages exhibited 2 and 3 clusters, respectively.

The analysis of the evolution of the sentiment of the posted tweets during the period since 2006 to 2022 exhibited interesting patterns. Bots, despite having shown a generally higher percentage of positive posts, exhibited a large number of posts with extreme values, a higher overall polarity and a higher proportion of neutral posts compared to human users. This sentiment characteristic remained relatively stable in the first four months of 2022 for both account types.

If we focus on the keyword analysis, especially that based on the centrality metric, we gain valuable information about the content of the messages. Human clusters show high similarity with each other (0.96). However, the bot clusters, especially cluster 1, exhibited the highest difference with the rest of clusters ($<0.86$). In spite of that, five words present in the aforementioned cluster 1 are common to all others.

This research not only expands our understanding of sustainability by analysing patterns of activity, relevant words and sentiment, but also provides practical information for distinguishing between human users and bots on Twitter. Below, we summarise certain limitations of the study carried out in this document, which were previously explained in detail.

Bot detection is a complex task, and the accuracy of bot detection tools is limited by the quality of the data and the algorithms used. In order to mitigate this, in reference to Botometer, several actions were carried out such as selecting only English tweets, choosing only users with public accounts, and establishing a threshold value for labelling in accordance with the values provided by other investigations. With respect to the analysis of sentiments, we use TextBlob due to its simplicity of use, its efficiency, and its processing times that are very appropriate for the volume of data used in this research.

Analysing human and bot activity patterns on Twitter was one of the primary objectives of this investigation, but the implications of the research findings extend beyond this social network. The high importance of sustainability issues in national and international contexts makes understanding the behaviour patterns of human and bot accounts very relevant. This knowledge would enable institutions and authorities, as policy makers and decision makers, to have a more effective influence in achieving more sustainable behaviour. Regarding sustainability matters, companies could also benefit from knowledge of human and bot activity patterns, as well as the sentiment analysis of messages posted on social networks in order to make their communication strategies more effective. Comprehending how bots can influence opinion on sustainability topics could also be of interest to citizens. All of the above allows us to raise awareness about the need to perform a critical analysis of the information published on social networks.

In future research, as a continuation of the investigation described in this document, we plan to implement a comparative sentiment analysis of the tweets utilising different sentiment analysers. Additionally, based on time series, we could implement sentiment prediction models. The procedures to be used could be some including artificial neural networks such as Long Short-Term Memory (LSTM), Adaptive Wavelet Neural Network (AWNN), Elman Recurrent Neural Networks (ERNN) or others such as autoregressive (AR), Autoregressive Integrated Moving Average (ARIMA), in addition to others. Tweets in Spanish could be used to expand the analysis here executed. The influence of bots on the perception of human users could also be explored through contagion models^63^.

## Methods

### Compression algorithms

In order to build a model to be used as one of the explanatory variables, the compression ratio of the raw user fingerprint and several compression algorithms such as gzip^64^, zlib^65^, bzip2^66^, lzma^67^ and smaz^68^ were used. The gzip method provided the best performance metrics, followed by zlib, bzip2, lzma. smaz algorithm exhibited the worst results, which seems to be because it was specially designed for short texts^69^. It must be noted that a large variability of digital fingerprint sizes are handled in this research.

For the purpose of comparing the methods, gzip, bz2, lzma, smaz, and zlib libraries in PYTHON, were used. They were applied considering the default parameters, in the gzip algorithm the parameters were: mode=’rb’ (it represents the mode of reading or writing the input/output file, ’r’:read, ’b’:binary) and compress level= 9 (it can take a value 0 (no compression), or any integer value in the range [1-9]. The higher the value, the higher the compression ratio and the lower the processing velocity. The default value is 9.).

zlib^65^ method was used considering $level=-1$ (this attribute can take the value 0 (no compression), -1 (trade-off between velocity and compression ratio), or any integer value in the range [1-9]. The higher the value, the more compression, and the slower the computation velocity. The default value is -1 (currently, this value is equivalent to 6)) and $wbits=MAX WBITS=15$ (it allow us to manage the window size which was utilised during the compression process as well as whether both header and trailer must have been included in the output. It can take values in the intervals [+9 to +15], [-9 to -15], [+25 to +31]. The default value es equal to 15.).

bzip2^66^ was applied with mode=’rb’ and compress level= 9 (these parameters have similar meaning to those explained in gzip algorithm).

lzma^67^ was carried taken: mode=’rb’ (this parameter has an analogous mining to that used in gzip algorithm), check=- 1 (it symbolises the integrity type examination to consider in the compressed information. It can take the values: CHECK$\_$NONE (-1): no integrity check is carried out; CHECK$\_$CRC32: a 32-bit Cyclic Redundancy Check is performed, CHECK$\_$CRC64: 64-bit Cyclic Redundancy Check is applied, CHECK$\_$SHA256: 256-bit Secure Hash Algorithm is carried out.).

smaz^68^ algorithm was implemented with mode=’r’, and buffering=1 (buffering refers to the mechanism of storing the input data prior to carrying out compression process. This parameter allows us to manage the application of a buffer policy, a value is equal to 1, means that a line is selected as buffering)).

All aforementioned algorithms above are described in the supplementary material document (see Sect. S2.1).

### Time series characterisation

For each user, in order to characterise the time series describing the number of daily tweets posted from 2006 to 2022, several parameters were calculated, which were mean, median, mode, standard deviation, maximum and minimum number of messages. Certain data regarding the seasonality of the series were also estimated utilising the Augmented Dickey Fuller test (ADF)^56^. They were maximum used lag order, which was estimated according to^70^, and *p*-*value*. It must be noted that with the objective of obtaining correct results in the test, an appropriate selection of lag order had to be carried out^58^.

### Building the dataset

In order to build a relevant dataset on sustainability, the keywords to carry out the selection of tweets had to be obtained, to do this task we gathered a small group of persons. If more than 35 keyword candidates were proposed, a clustered and affinity diagram was applied. After this, the obtained keywords were sorted by applying the multiple voting system. Through this procedure, those words that reinforce each other were linked. Finally, the following keywords were chosen for downloading tweets: sustainable agriculture, sustainable food, renewable energy, green urban, sustainable transport, pollution, sustainable city, and sustainable industry.

The reason for reducing the number of words from 35 to 8, as described above, was determined by the keyword selection system itself. The used election method has shown optimal results in other studies^9,11^. The number of words to choose was also conditioned by the feasibility of obtaining, processing and analysing the data. Downloading and processing the tweets from 2006 to 2022 containing these 8 keywords took approximately 12 months, resulting in a volume of 233 GB of raw data and a similar magnitude of processed data. Using 35 words would have lengthened downloading and processing times by involving a much larger volume of data, which could have compromised the novelty of the research. 8 is considered an appropriate number of words to achieve an optimal compromise between the amount of information downloaded from Twitter and the processing times needed to conduct the investigation.

In the same way to^9^,^10^, and^11^, twarc2 software was utilised to download the Twitter messages. Then, T-Hoarder tool^71^ was applied (see Supplementary Material Document, Sect. S1) for formatting the messages provided by Twarc2 from JSON to csv format. The procedure to build the dataset used in this study can be summarized as follow:

- 40,000 users were randomly selected among all those who at some time sent a tweet with any of the aforementioned keywords. Next, all their messages (tweets, replies, retweets and quotes) from 2006 to 2022 were then downloaded. It must be noted that 2006 was the year in which Twitter was created. From 40,000 users only those with a public account were taken into consideration. In our study we considered 38,615 users that posted 96,252,871 tweets.
- In addition to the above, all posted tweets in 2022, including the previously mentioned keywords, were downloaded. 684,090 tweets were utilised, which corresponded to 259,170 users.

### Preparation of tweets for analysis

For each of the 38,615 selected users, a footprint was built. All messages corresponding to a user were coded according to their type: tweet as ’A’, retweet as ’T’, reply as ’C’ and quote as ’G’, resulting in a string of variable size. This footprint can be considered an attribute of each user, similar to human DNA.

In order to build a model that allows us to distinguish between humans and bots, the digital footprint was compressed utilising the algorithms described in Sect. "Compression algorithms".

In conjunction with the above, in order to generate an indicator that revealed whether the user was a human or a bot, Botometer API was utilised^72^. This information was used to build the model. Botometer API provided a value in [0,1] range, based on different bot identification methodologies. We assume as bots those users which were characterised with a value $>0.9$. Although other values were tested, this magnitude, as we have already mentioned, provided a percentage of bots in line with that indicated by existing pieces of research^62^.

Additionally, the text of each tweet was also processed. In the same way to^9^,^10^, and^11^ various replacements were made: all URLs were changed by the term “LINK”, all users' mentions were modified the term “USER”, hashtags were modified by term “HASHTAG”. Emoticons were transformed to their meaning in text. In addition to the above we also carried out various modifications on the text of the tweet, which were: elimination of conjunctions, terms with a size lower than 3 characters and punctuation symbols. We also changed the figures corresponding to the same term by its lemma. We also corrected the misspelt words and transformed the text to lowercase^9^,^10^, and^11^. The text of the tweets corresponding to 2022, was also identically processed.

### Building the model

As explained in Sect. "Building the model", in order to detect possible differences between tweets coming from bot and human users, a model to identify the users’ categorisation was implemented. The model used several features (according to the selection alternative chosen) and applied various mathematical procedures: Random Forest (*RFM*), Generalised Linear (*GLM*) and Support Vector Machine (*SVM*) algorithms.

Prior to the construction of the model it was necessary to check the correlation between input variables. The Pearson or Spearman method would be applied depending on whether the variables were normally distributed or not, which was examined using the Anderson-Darling test^73^. The following hypotheses were utilised, with a significance level equal to 0.05:

(i) $H_{0}$: “The sample derived from a normal distribution”
(ii) $H_{a}$: “The sample did not derive from a normal distribution”.

If *p*-$value<0.05$, $H_{0}$ would be rejected.

Those variables that exhibited a correlation of more than 0.75 with another variable were eliminated.

Similarly to^10,74,75^, in order to build the model, a cross-validation procedure was performed in which *NF* folds were used. The model was trained *NF* times, where each time 1 fold was taken as a test set, and each of the remaining $NF-1$ folds were utilised as a training set. To estimate the model appropriateness, the mean of an estimated metric (*ESTMET*) was calculated^75^:

2 $$\begin{aligned} <ESTMET>=\frac{1}{NF} \sum _{i=1} ^{i=NF} ESTMET_{i} \end{aligned}$$

*ESTMET*  represents Accuracy, Sensitivity, Specificity, and kappa. A separate end estimation of the aforementioned metrics was also computed utilising the validation sets. *NF* took a value equal to 5. In order to execute the CV process 80% of the samples were used as training and 20% as validation set.

A short description of the *GLM*, *RFM* and *SVM* has been included in the Supplementary Material Document (Sect. S2.2).

### Account typologies

With the aim of describing the activity corresponding to each user, related to tweeting, retweeting, replying, and quoting the following attributes were analysed:

- Seasonality of the time series. Parameters related to the Augmented Dickey-Fuller test, which allowed us to examine the existence of this property in the user’s activity, these were: (i) Maximum used lag order, computed as^70^, where several models with different lag orders are fitted and the model with the lowest AIC value is selected. The maximum lag order is defined as the order of the model with the lowest AIC value. (ii) obtained *p*-*value*.
- For each user, various statistical magnitudes such as number, mean, median, mode, standard deviation, as well as maximum, and minimum number of daily tweets were considered.

In order to examine if there are differences between the above-mentioned variables for humans and bots, the following procedure was performed.

- The normality of distributions of each variable was examined utilising the D’Agostino test^76^. A significance level equal to 0.05 was considered.
- If normality existed, the existence of homoscedasticity in each variable should be checked through the Breusch-Pagan test. A significance level equal to 0.05 was utilised.
- If normality and homoscedasticity existed, the analysis of variance (ANOVA) method should be executed.
- By contrast, if there was no normality or homoscedasticity in the distributions, the Kruskal-Wallis test would be computed.

### Activity patterns

With the goal of detecting behavioural patterns, in both humans and bots, we use time series clustering, which is an unsupervised data mining technique that groups data points based on their similarity.

For each type A, C, G, and T messages, the parameters indicated in the previous section were considered. Accordingly, we applied K-Means method to a set of data points *SDP*: $x_{i}, x_{2}, x_{n}$ in which $\mathbf {x_{i}} = (x_{i1}, x_{i2},..., x_{id})$ symbolises a vector in $R^{d}$, and then we get the data distributed into *k* clusters. Each cluster is represented by a centroid. Given a *k* value, K-Means method operates as followed^77,11^:

- Step 1: In order to represent the first centroids, *k* data points are picked at random
- Step 2: Each data point is assigned to the nearest centroid.
- Step 3: Considering the present cluster memberships, we compute the centroids again
- Step 4: In case the convergence criterion is not met, we will repeat steps 2 and 3.

The following convergence criterion is considered^77,11^:

- There is no or a very small re-designation of data points to different clusters that exists, or
- There is no modification of centroids or there is a minimal change.
- There is a very small reduction in the sum of squared errors (SSE).
  3 $$\begin{aligned} SSE=\sum _{j=1}^{k}\sum _{x \in C_{j}}d(x,cen_{j})^{2} \end{aligned}$$
- $C_{j}$ Where ^77,11^ the $j^{th}$ cluster
- $cen_{j}$ is the centroid of cluster $C_{j}$ (the mean vector of all the data points in $C_{j}$),
- $d(x, cen_{j})$ is the Euclidean distance between data point *x* and centroid $cen_{j}$.
- *k* is the number of clusters.

For human and bot users, the optimal number of clusters is estimated utilising the Silhouette and Dunn indexes, which are described in the Supplementary Material Document (see Sect. S2.3).

### Sentiment analysis

The analysis of sentiment and subjectivity of each tweet was carried out using the Python TextBlob^78^ library. This library examines the text of each tweet providing a value for the sentiment and the subjectivity of the tweet in the ranges [-1,1] and [0,1], respectively. This analysis is based on a word lexicon in which each word is described by its positivity /negativity as well as by its subjectivity (subjectivity determines the personal opinion that the text of the tweet contains. The higher the subjectivity, the more personal opinion than objective information there is^9^,^11^). As we have already explained, the sentiment and subjectivity corresponding to each tweet are calculated by averaging the sentiment and subjectivity for all words included in the tweet. Only English-language tweets were used.

It should be noted that although recent methods such as BERT have proven to be very appropriate for the analysis of complex texts^79–83^, we have used the TextBlob library because in addition to its simplicity of use, it has demonstrated optimal results^9,11, 84–87^. In this research, as mentioned above, the average sentiment of each tweet was calculated from the sentiment shown for each word in the text of each processed tweet. Due to this and the fact that we handle a large volume of data, with Textblob being faster and less computationally expensive (CPU and GPU) than BERT^88^, we have chosen TextBlob as the sentiment analyser.

### Keywords analysis

A keyword analysis was carried out using two procedures, which allowed us to compare the results obtained with each method.

Method 1. In this procedure, once the tweets were processed as indicated in Sect. "Preparation of tweets for analysis", a word count was performed, and the most repeated words were considered as the most important.

Method 2. In this approach a similar procedure to the one described in^61^ was performed. It is named *TKG*, which is based on the creation of word graphs, , and examines them utilising certain centrality metrics.

Once the tweets were processed as indicated in Sect. "Preparation of tweets for analysis", they were sorted into groups of 30 elements each (this value was taken considering an adequate compromise between network size and computational speed). After this, two types of graphs were constructed from the words included in each tweet. The type 1 graph connected two words, provided one followed the other in the text of tweet $TKG_1$. The type 2 graph connected all words that were in the same group of 30 elements ($TKG_2$). It must be noted that, in the type 1 graph, in order to establish the weights of the links between two nodes *i*, *j*, two mechanisms were used: (mechanism 1) the co-occurrence frequency was taken as the link weight, this is, $w_{ij} = f_{ij}$, and (mechanism 2) the inverse co-occurrence frequency was considered as weight, and this is, $w_{ij} = 1/f_{ij}$. In the type 2 graph only the mechanism 1 was taken into consideration.

The following centrality metrics were considered:

- Degree centrality (*DC*) of a node *i*, which is described as:^89^
  4 $$\begin{aligned} DC_{i} =deg_i \end{aligned}$$
  where $deg_i$ symbolises the number of links connected to the node $i$, and
- Closeness centrality (*CC*) of a node *i*, which is defined as:^90^
  5 $$\begin{aligned} CC_{i}=\frac{1}{\sum _{ij\in V}d_{ij}} \end{aligned}$$
  where $V$ is the set of nodes and $d_{ij}$ is the shortest path between the nodes $i$ and $j$.

## Software programs

Several functionalities were coded in R language, such as: (1) Construction of the model, where the caret and vip packages were used. (2) Cluster and tendency analyses were performed utilising the NbClust and clustertend packages. (3) Execution of statistical tests, in which the nortest and tseries packages were used. (4) Keyword analysis (network building and calculation of centrality metrics), was carried out using the igraph and NLP packages (5) Plotting graphs was implemented using the ggplot2.

Other various funtionalities were also implemented in PYTHON language, which were: (1) Compression algorithms gzip, bzip2, zlib, lzma and smaz, where the packages: gzip, bz2, zlib, lzma and smaz were utilised. (2) Emoji analysis, where emoji and demoji libraries were used to select and replace the emojis by text. (3) Keywords examination, where re package was utilised to correct misspellings as well as to find and extract the hashtags from each tweet. For the removal of irrelevant words, the nltk package was used in conjunction with the stopwords package. For splitting text into tokens and lemmatisation of words, the tokenize and wordnet extensions of the nltk package were used.

### Ethics approval

The research complied with all the relevant national regulations. We only used public tweets.

### Consent to participate

There is consent from the authors to participate in the manuscript.

### Supplementary Information

Supplementary Information.

## Data Availability

The data utilised to support the findings of this research are available from the corresponding author upon request.

## References

[1] Sustainable Development Goals. https://www.un.org/en/academic-impact/sustainability

[2] United Nations (w.d.) Department of Economic and Social Affairs Sustainable Development. Transforming our world: the 2030 Agenda for Sustainable Development. https://sdgs.un.org/2030agenda.

[3] Cabelkova I, Smutka L, Mareš D, Ortikov A, Kontsevaya S (2023). Environmental protection or economic growth? The effects of preferences for individual freedoms. Front. Environ. Sci..

[4] Wu L, Qing C, Jin S (2022). Environmental protection and sustainable development of enterprises in China: The moderating role of media attention. Front. Environ. Sci..

[5] Arbatani T, Labafi S, Robati M (2016). Effects of social media on the environmental protection behaviour of the public (Case study: Protecting Zayandeh-rood river environment). Int. J. Environ. Res..

[6] Yu B, Ruxin Y (2023). Visualizing environmental management: Corporate environmental images information disclosure and idiosyncratic risk. Environ. Impact Assess. Rev..

[7] DMR: Twitter Statistics, User Count and Facts. https://expandedramblings.com. (Accessed 01 April 2023) (2023).

[8] Buccafurri F, Lax G, Nicolazzo S, Nocera A (2015). Comparing Twitter and Facebook user behavior: Privacy and other aspects. Comput. Hum. Behav..

[9] Mouronte ML, Ceres J, Columbrans A (2023). Analysing the sentiments about the education system trough twitter. Educ. Inf. Technol..

[10] Mouronte ML, Subirán M (2022). Modeling the interaction networks about the climate change on twitter: A characterization of its network structure. Complexity.

[11] Mouronte-López ML, Subirán M (2022). What do twitter users think about climate change? Characterization of twitter interactions considering geographical, gender, and account typologies perspectives. Weather Clim. Soc..

[12] Batrinca B, Treleaven PC (2015). Social media analytics: A survey of techniques, tools and platforms. AI Soc..

[13] Gómez, L. M., Sustainability and CSR Views on Twitter: A Pilot Study Analysis. In 10th International Conference on Corporate Social Responsibility. https://ssrn.com/abstract=1966308 (2011).

[14] Pons A, Rius J, Vintró C, Gallart A (2022). Analysis of twitter posts for evaluation of corporate social responsibility in the leather industry. J. Eng. Fibers Fabr..

[15] Salvatore C, Biffignandi S, Bianchi A (2022). Corporate social responsibility activities through twitter: from topic model analysis to indexes measuring communication characteristics. Soc. Indic. Res..

[16] Johnson TF, Greenwell MP (2022). Are companies using Twitter to greenwash and hide bad environmental performance?. Energy Ecol. Environ..

[17] Patuelli A, Saracco F (2023). Sustainable development goals as unifying narratives in large UK firms’ Twitter discussions. Sci. Rep..

[18] Shao C, Hui P-M, Wang L, Jiang X, Flammini A, Menczer F, Ciampaglia G (2018). Anatomy of an online misinformation network. PLoS One.

[19] Vosoughi S, Roy D, Aral S (2018). The spread of true and false news online. Science.

[20] De Clerck B, Rocha LEC, Van Utterbeeck F (2022). Maximum entropy networks for large scale social network node analysis. Appl. Netw. Sci..

[21] Bovet A, Morone F, Makse H (2018). Validation of twitter opinion trends with national polling aggregates: Hillary Clinton vs Donald Trump. Sci. Rep..

[22] Carballosa A, Mussa-Juane M, Muñuzuri A (2020). Incorporating social opinion in the evolution of an epidemic spread. Sci. Rep..

[23] Schuchard R, Crooks A, Stefanidis A, Croitoru A (2019). Bots fired: Examining social bot evidence in online mass shooting conversations. Palgrave Commun..

[24] Radicioni T, Saracco F, Pavan E, Squartini T (2021). Analysing twitter semantic networks: The case of 2018 Italian elections. Sci. Rep..

[25] Borondo J, Morales A, Losada González JC, Benito R (2012). Characterizing and modeling an electoral campaign in the context of twitter: 2011 Spanish presidential election as a case study. Chaos.

[26] Lim SBPJ (2022). Opinion amplification causes extreme polarization in social networks. Sci. Rep..

[27] Martin-Gutierrez S, Losada González JC, Benito R (2022). Multipolar social systems: Measuring polarization beyond dichotomous contexts. Chaos Solitons Fractals.

[28] Morales A, Borondo J, Losada González JC, Benito R (2015). Measuring political polarization: Twitter shows the two sides of Venezuela. Chaos.

[29] Shao C, Ciampaglia G, Varol O, Flammini A, Menczer F, Yang K-C (2018). The spread of low-credibility content by social bots. Nat. Commun..

[30] Wang W, Chen X, Jiang S, Wang H, Yin M, Wang P (2020). Exploring the construction and infiltration strategies of social bots in Sina microblog. Sci. Rep..

[31] Caldarelli G, De Nicola R, Del Vigna F, Petrocchi M, Saracco F (2020). The role of bot squads in the political propaganda on twitter. Commun. Phys..

[32] Mønsted B, Sapiezynski P, Ferrara E, Lehmann S (2017). Evidence of complex contagion of information in social media: An experiment using twitter bots. PLoS One.

[33] Dunn A, Surian D, Dalmazzo J, Rezazadegan D, Steffens M, Dyda A, Leask J, Coiera E, Dey A, Mandl K (2020). Limited role of bots in spreading vaccine-critical information among active twitter users in the united states: 2017–2019. Am. J. Public Health.

[34] Pasricha, N., Hayes, C. Detecting bot behaviour in social media using digital DNA compression. In: Irish Conference on Artificial Intelligence and Cognitive Science (2019). https://www.semanticscholar.org/paper/Detecting-Bot-Behaviour-in-Social-Media-using-DNA-Pasricha Hayes/7f88a79589f980baae72b4e0a627d85fb0aa6e66

[35] Shevtsov A, Tzagkarakis C, Antonakaki D, Ioannidis S (2022). Identification of twitter bots based on an explainable machine learning framework: The US 2020 elections case study. Proc. Int. AAAI Conf. Web Soc. Media.

[36] Chavoshi, N., Hamooni, H. & Mueen, A. *Identifying correlated bots in twitter*. 10.1007/978-3-319-47874-6_2 (2016).

[37] Fontanelli, O., Venegas, A. & Mansilla, R. Analyzing time series activity of Twitter political spambots. 10.48550/arXiv.2105.12734 (2021).

[38] Mazza, M., Cresci, S., Avvenuti, M., Quattrociocchi, W., & Tesconi, M. RTbust: Exploiting Temporal Patterns for Botnet Detection on Twitter. In: *Proc. of the 10th ACM Conference on Web Science*, 183-192 (2019). 10.1145/3292522.3326015

[39] Chavoshi, N., Hamooni, H. & Mueen, A. *Temporal Patterns in Bot Activities*. 10.1145/3041021.3051114 (2017).

[40] Loef B, Wong A, Janssen N, Strak M, Hoekstra J, Picavet S, Boshuizen H, Verschuren W, Herber G-C (2022). Using random forest to identify longitudinal predictors of health in a 30-year cohort study. Sci. Rep..

[41] Stephan J, Stegle O, Beyer A (2015). A random forest approach to capture genetic effects in the presence of population structure. Nat. Commun..

[42] Li J, Chen X, Huang Q, Wang Y, Xie Y, Dai Z, Zou X, Li Z (2020). Seq-symrf: A random forest model predicts potential mirna-disease associations based on information of sequences and clinical symptoms. Sci. Rep..

[43] Rustam, Z., Saragih, G.S.: Predicting bank financial failures using random forest. In: 2018 International Workshop on Big Data and Information Security (IWBIS), pp. 81–86 (2018). 10.1109/IWBIS.2018.8471718

[44] Jiang L, Zheng Z, Fang H, Yang J (2021). A generalized linear mixed model association tool for biobank-scale data. Nat. Genet..

[45] Jiang M, Wang X (2021). Research on intelligent prediction method of financial crisis of listed enterprises based on random forest algorithm. Secur. Commun. Netw..

[46] Khaiwal R, Rattan P, Mor S, Nath A (2019). Generalized additive models: Building evidence of air pollution, climate change and human health. Environ. Int..

[47] Schneider J, Vlachos M (2023). A survey of deep learning: From activations to transformers. ArXiv.

[48] Vaswani, A., Shazeer, N., Parmar, N., Uszkoreit, J., Jones, L., Gomez, A. N., Kaiser, L., & Polosukhin, I. Attention is all you need. In 31st Conference on Neural Information Processing Systems (NIPS 2017), Long Beach, CA, USA. https://proceedings.neurips.cc/paper_files/paper/2017/file/3f5ee243547dee91fbd053c1c4a845aa-Paper.pdf (2017).

[49] Kumar A, Yuqing Z, Gandhi CP, Kumar R, Xiang J (2020). Bearing defect size assessment using wavelet transform based deep convolutional neural network (DCNN). Alex. Eng. J..

[50] Nayak, R., Sethy, A., Patra, P. & Sahoo, D. Transform based approach for handwritten character and numeral recognition a comprehensive approach. In International Conference on Artificial Intelligence in Manufacturing & Renewable Energy 2019. https://papers.ssrn.com/sol3/papers.cfm?abstract_id=3708702 (2019).

[51] Domingo L, Grande M, Borondo F, Borondo J (2023). Anticipating food price crises by reservoir computing. Chaos Solitons Fractals.

[52] Yang C, Harkreader R, Gu G (2013). Empirical evaluation and new design for fighting evolving twitter spammers. IEEE Trans. Inf. Forensics Secur..

[53] MacKay DJC (2003). Information Theory, Inference and Learning Algorithms.

[54] Rauchfleisch A, Kaiser J (2020). The False positive problem of automatic bot detection in social science research. PloS One.

[55] Chu Z, Gianvecchio S, Wang H, Jajodia S (2012). Detecting automation of twitter accounts: Are you a human, bot, or cyborg?. IEEE Trans. Dependable Secur. Comput..

[56] Cheung Y-W, Lai KS (1995). Lag order and critical values of the augmented dickey-fuller test. J. Bus. Econ. Stat..

[57] Mushtaq R (2011). Augmented dickey fuller test. Econom. Math. Methods Program. eJ..

[58] Cavaliere G, Phillips PCB, Smeekes S, Taylor AMR (2015). Lag length selection for unit root tests in the presence of nonstationary volatility. Econom. Rev..

[59] Ostertagova E, Ostertag O, Kováč J (2014). Methodology and application of the Kruskal-Wallis test. Appl. Mech. Mater..

[60] Sitikhu, P., Pahi, K., Thapa, P. & Shakya, S. A Comparison of Semantic Similarity Methods for Maximum Human Interpretability. 10.1109/AITB48515.2019.8947433 (2019).

[61] Abilhoa WD, de Castro LN (2014). A keyword extraction method from twitter messages represented as graphs. Appl. Math. Comput..

[62] Varol O, Ferrara E, Davis C, Menczer F, Flammini A (2017). Online human-bot interactions: Detection, estimation, and characterization. Proc. Int. AAAI Conf. Web Soc. Media.

[63] Bandari, R., Asur, S., Huberman, B.A. Pulse: real-time event tracking using social media. In 2016 IEEE/ACM International Conference on Advances in Social Networks Analysis and Mining (ASONAM), 1025–1028 (IEEE, 2016).

[64] Deutsch. L. P. GZIP file format specification version 4.3. https://www.rfc-editor.org/info/rfc1952 (1996).

[65] Adler, M. Zlib Compressed Data Format Specification version 3.3. RFC 1950. https://www.ietf.org/rfc/rfc1950.txt (1996).

[66] Seward, J. https://www.sourceware.org/bzip2/docs.html (1996).

[67] Collin, L. & Pavlov, I. LZMA SDK (Software Development Kit) 9.22 beta: Description of LZMA compression. https://www.7-zip.org/sdk.html (2015).

[68] Antirez, S. SmaZ - a short string compression library. In Proceedings of the 3rd Annual Redis Conference. https://antirez.com/misc/smv6.pdf (2012).

[69] Smaz documentation. https://docs.rs/smaz/latest/smaz

[70] Schewert GW (1989). Test for unit roots: A Monte Carlo investigation. J. Bus. Econ. Stat..

[71] Congosto M, Basanta-Val P, Sanchez-Fernandez L (2017). T-hoarder: A framework to process twitter data streams. J. Netw. Comput. Appl..

[72] Davis, C., Varol, O., Ferrara, E., Flammini, A., Menczer, F.: Botornot: A system to evaluate the veracity of online identities. In Proc. of the 25th International Conference Companion on World Wide Web, 273–274 (ACM, 2016).

[73] Anderson, T. Anderson-darling tests of goodness-of-fit, 52–54 (2011). 10.1007/978-3-642-04898-2_118

[74] Mouronte ML (2021). Modeling the public transport networks: A study of their efficiency. Complexity.

[75] Mouronte-López ML, Gómez J (2023). Exploring the mobility in the Madrid Community. Sci. Rep..

[76] D’Agostino, R. *Normality tests: Overview*. 10.1002/9781118445112.stat05920 (2014).

[77] Ullman, S., Harari, T.P.D., Zysman, D., Seibert, D. 9.54 class 13 unsupervised learning clustering. **3**, (2014).

[78] TextBlob (w.d) TextBlob (w.d.) TextBlob: Simplified Text Processing. https://textblob.readthedocs.io/en/dev/

[79] Alaparthi S, Mishra M (2021). BERT: A sentiment analysis odyssey. J. Mark. Anal..

[80] Sun, C., Huang, L. & Qiu, X. Utilizing BERT for Aspect-Based Sentiment Analysis via Constructing Auxiliary Sentence. NAACL-HLT (1), 380-385 (2019). https://arxiv.org/abs/1903.09588.

[81] Batra, H., Punn, N. S., Sonbhadra, S. K., & Agarwal, S. BERT-based sentiment analysis: A software engineering perspective. In Database and Expert Systems Applications, 138-148. 10.1007/978-3-030-86472-9_13.

[82] Marcacini, R. & Silva, E. Aspect-based sentiment analysis using BERT with disentangled attention. 10.52591/lxai2021072410 (2021).

[83] M. Munikar, S. Shakya and A. Shrestha, Fine-grained sentiment classification using BERT. In 2019 Artificial Intelligence for Transforming Business and Society (AITB), 1-5 (2019). 10.1109/AITB48515.2019.8947435.

[84] Chandrasekaran G, Hemanth J (2022). Deep learning and TextBlob based sentiment analysis for coronavirus (COVID-19) using twitter data. Int. J. Artif. Intell. Tools.

[85] Aljedaani W, Rustam F, Mkaouer MW, Ghallab A, Rupapara V, Washington P, Lee E, Ashraf I (2022). Sentiment analysis on Twitter data integrating TextBlob and deep learning models: The case of US airline industry. Knowl. Based Syst..

[86] Chaudhri AA, Saranya SS, Dubey S (2021). Implementation paper on analyzing COVID-19 vaccines on twitter dataset using Tweepy and text blob. Ann. Rom. Soc. Cell Biol..

[87] Susrama G, Mandenni N, Fachrurrozi M, Ilham Pradika S, Manab K, Sasmita N (2021). Twitter sentiment analysis as an evaluation and service base on python Textblob. IOP Conf. Ser. Mater. Sci. Eng..

[88] Gichere, F. *Sentiment Analysis of App Reviews: A Comparison of BERT, spaCy, TextBlob, and NLTK*. https://francisgichere.medium.com/sentiment-analysis-of-app-reviews-a-comparison-of-bert-spacy-textblob-and-nltk-9016054d54dc (2023).

[89] Nieminen J (1974). On the centrality in a graph. Scandinavian J. Psychol..

[90] Wasserman, S., & Faust, K. Social network analysis: Methods and applications. Cambridge university press (1994).

