# Supplementary material for: Patterns of human and bots behaviour on Twitter conversations about sustainability
Source: Sci Rep. 2024 Feb 8;14:3223. doi: 10.1038/s41598-024-52471-z (PMC10853507; doi:10.1038/s41598-024-52471-z)
Supplement: Supplementary file 1 — Supplementary Information. [file 41598_2024_52471_MOESM1_ESM.pdf]

Patterns of Human and Bots Behaviour on Twitter Conversations about Sustainability (Supplementary Material)

Mary Luz Mouronte-López[1*], Javier Gómez Sánchez-Seco[1,2] and Rosa M. Benito[2]

[1*]Higher Polytechnic School, Universidad Francisco de Vitoria, Carretera Pozuelo a, Av de Majadahonda, Km 1.800, Pozuelo de Alarcón, 28223, Madrid, Spain.
[2]Grupo de Sistemas Complejos, Escuela Técnica Superior de Ingeniería Agronómica, Alimentaria y de Biosistemas, Universidad Politécnica de Madrid, Avda. Puerta de Hierro 2-4, Madrid, 28040, Madrid, Spain
.

*Corresponding author(s). E-mail(s): maryluz.mouronte@ufv.es; Contributing authors: javier.gsanchezseco@alumnos.upm.es; rosamaria.benito@upm.es;

Keywords: sustainability, Twitter, modelling, cluster analysis, bot, sentiments

S1 Resources

`Botometer`

`Botometer` is a software tool that uses a trained machine learning algorithm to provide a score calculated based on the activity of a twitter account. Higher scores mean that the account has more bot-like activity (the score is in the range [0,1]) [1].

`Twarc2`

`Twarc2` is a `PYTHON` tool and library, whose functionality makes it possible to download tweets with specific content in JSON format [2].

`T-Hoarder`

In this research, similarly to [3], [4], and [5] `T-Hoarder` tool is used to format the file provided by `twarc2` to plain text. In this text format the information for each tweet is stored on a single line with tab-separated fields [6], [4], [3], [5] the following parameters are included:

`id tweet`: A sequentially assigned increasing number that identifies each interaction type.

`date`: Date and time GMT corresponding to the time the interaction took place.

`author`: Username of the tweet author that is associated with the @.

`text`: Text included in the interaction.
`app`: Application used to publish the interaction.

`id user`: A unique increasing number assigned by Twitter to each user upon logging in.

`followers`: Number of users that follow the author's interaction.

`following`: Numbers of users the author's interaction follows.

`stauses`: Author's number of previously published tweets.

`location`: Location registered by the user in his/her profile.

`urls`: If the interaction contains a URL, it is stored in this field. Otherwise, it stores a null value.

`geolocation`: Coordinates that identify the user's location if the interaction is geotagged.

`name`: User provided name.

`description`: User provided description (also known as *bio*).

url_media: If the interaction contains multimedia information, it stores the URL.

`type media`: Type of multimedia information.

quoted: If the interaction is quoted by the user. It can be Yes or None.

`relation`: Tweet interaction type (None as tweet, retweet, reply or quote).

`replied_id`: Number identifying the tweet being replied to.

`user replied`: Screen name of the tweet's author that is replied.

`retweeted_id`: Number identifying the tweet that is retweeted.

`user retweeted`: Screen name of the tweet's author that is retweeted.

quoted_id: Number identifying the tweet that is quoted.

`user quoted`: Screen name of the tweet's author that is quoted.

`first HT`: First hashtag that appears in the interaction.

`lang`: Language of the interaction.

`created_at`: Date and GMT hour of the account creation.

`verified`: If the author has verified their profile. It can take the values true or false.

avatar: URL containing the user's avatar image in jpg format.

`link`: URL containing the interaction

`RTs`: Number of retweets the interaction has.

`replies`: Number of replies the interaction has.

`quotes`: Number of quotes the interaction has.

`fav`: Number of favorites the interaction has.

S2 Methods

S2.1 Compression algorithm

`Huffman Algorithm`

This algorithm assigns bit codes (with different length) to each character included in a text. The procedure is as follows [7], [8], [9], [10], [11]:

(i) To count how many times each character appears in the text to be compressed. Then, to build a linked list with the characters and their frequencies.

(ii)To order the list from lowest to highest frequency.

(iii) To convert each item in the list into a tree.

(iv) To merge all these trees into a single tree, according to the following process [7], [8], [9], [10], [11]:

(a) With the first two trees a new one is built, in which each of the processed trees symbolises a branch. (b) To include the frequencies of each branch as elements in the new tree. (c) According to the sum obtained by adding up the frequencies of each branch, to insert the new tree in the appropriate place in the list. Successively, until all nodes are processed, the next node is taken, and the same operation is performed.

(v) The corresponding codes are assigned to each branch, e.g. the branches on the left zeros, and on the right ones.

Example

As an example, the following text: `"TTTTAAGAAACCCACTA"` is considered, which corresponds to the footprint of a user.

(i) The number of times each character appears is counted (it is included in brackets). A linked list is built (see Figure S1)

'T ' (4), 'A' (7), 'G'(1), 'C' (4)

Fig. S1 In example corresponding to Huffman Algorithm, step i

(ii) The above sequence is ordered from lowest to highest frequency (see Figure S2)

'G ' (1), 'T' (4), 'C' (4), 'A' (7)

Fig. S2 In example corresponding to Huffman Algorithm, step ii

(iii) Each element in the sequence is considered as a tree (see Figure S3)

'G ' (1)->'T' (4)->'C' (4)->'A' (7)

Fig. S3 In example corresponding to Huffman Algorithm, step iii

(iv) First two nodes (trees) are merged into a new tree. The frequencies of the two nodes are added, the new tree is placed in the appropriate location (see Figure S4)

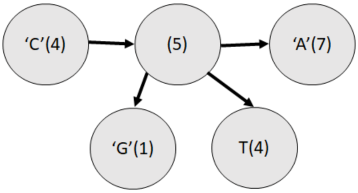

Fig. S4 In example corresponding to Huffman Algorithm, step iv

(continuation step iv) The previous step is repeated, as long as the list contains more than one element. Finally, the tree described in Figure S5 is obtained

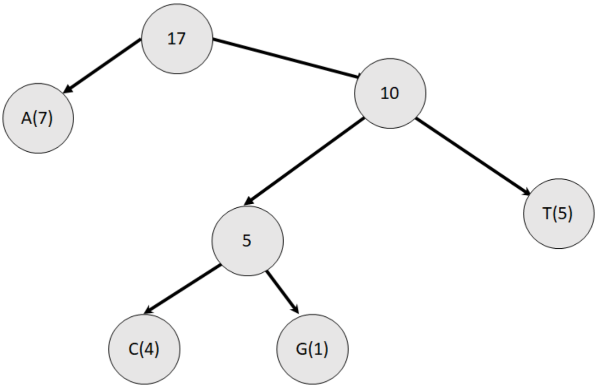

Fig. S5 In example corresponding to Huffman Algorithm, step v

(step v) The codes are assigned, considering as a criterion, that the left branches are '0', and the right branches are '1'. 'A':0;'C':100;'G':101;'T':11

`gzip` Algorithm

Firstly, the method uses the `lz77` mechanism, and then the obtained result is compressed through Huffman coding (see previous section) [12], [13]. As explained [13], the `Lz77` method changes the repeated strings by a tuple (d, l), in which d symbolises the distance in bytes and l denotes the length of the repeated string, respectively. As an example, the text: 'uvndeuvn' is compressed to: 'uvnde(5,3)'.

At each point within the text, `lz77` tries to find the longest string that is included within the immediate previous window, which has assigned a specific size. If such a string is found, `lz77` changes the current string with a pointer to that occurrence. If no repetition longer than 2 bytes is found, then these bytes are not compressed[13]. It must be noted that a character occupies 1 byte.

Utilising a dictionary, the Huffman coding allocates to symbols from a given alphabet a variable-size codeword (coded symbol). As an example, an appropriate Huffman coding for a four-character "alphabet" with four variable-length codes, could be: 'A': 01; 'C': 10; 'T': 110; 'G': 1111. For this, "alphabet", a possible tree representation is shown in Figure S6.

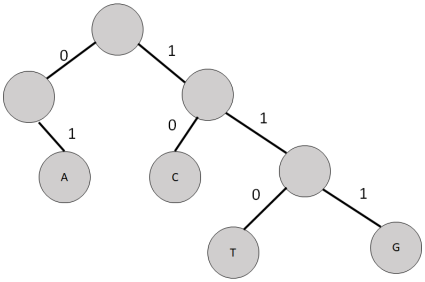

Fig. S6 As an example, for 'A': 01; 'C': 10; 'T': 110; 'G': 1111, Huffman code represented as a tree

Regarding the decoding process, it begins at the top of the tree, reading a bit (which can be '0' or '1'). If it is '0', the process continues along the left branch of the tree. By contrast, if the bit is 1, the process is carried out with the right-branch. If a leaf node is reached the process ends. The process returns the symbol and come back to the top of the tree.

`zlib` Algorithm

Similar to `gzip` algorithm [14], [15] the `zlib` method utilises lz77 [16] and Humann procedures. It can be described as follows:

- (i) Initialisation: a sliding window of size W is initialised, which moves along the input data in a sequential manner.
- (ii) Repeating pattern search: the algorithm searches for existing repeating patterns within the sliding window using the `lz77` algorithm. If a repeating pattern is found, it is replaced by a reference to the previous occurrence of the pattern.

  `Huffman` coding: the algorithm applies the Huffman coding (see corresponding section)
- The compressed data are obtained [15].

Example

As an example the following text is considered `"TTTTAAGAAACCCACTA"`, which is the digital footprint of a user.

A sliding window size with value 4 is taken. The algorithm begins initialising the sliding window and moving it over the input data. The first repeated pattern found is "TTT", which is replaced with a reference to the previous occurrence of the pattern (offset=3, length=3), followed by "A". The sliding window then moves to the next position, and the sequence "TTA" is found. This sequence is encoded as a reference to the previous occurrence of the pattern (offset=2, length=3), followed by "G". The algorithm continues in this way until the entire input data has been processed.

`lzma` Algorithm

This algorithm uses the `lz77` method and Marlov's [17] chains to model the context [18], [19]. It works by dividing the input data into blocks and compressing each block separately. The algorithm consists of three steps, which are described below.

Initialisation stage: in this phase, the dictionary is initialised.

Matching stage: in this phase, the algorithm looks up for the longest match between the current position and the dictionary entries. The following formula is utilised:

$$\text{match\_length} = \max\{l : X[l..] \text{ is a prefix of some } D[i..]\}$$

Where:

X symbolises the current position in the input data,

D represents the dictionary entries.

l denotes the match length.

i symbolises the dictionary entry index.

Encoding stage: in this phase, the algorithm encodes the match length, distance, and the current symbol utilising an arithmetic coding based on a probability model. The following formulas are used [19]:

$$encoded_{ML} = encode(ML \mid p_{ML})$$
$$encoded_{DIST} = encode(DIST \mid p_{DIST})$$
$$encoded_{SYM} = encode(SYM \mid p_{SYM})$$

Where:

ML denotes the match length,

$DIST$ symbolises the match distance,

SYM represents the current symbol,

$(x \mid p)$ denotes the arithmetic coding corresponding to x symbol, which was obtained using the probability distribution p,

p_{ML}, p_{DIST}, and p_{SYM} symbolise the probability models, which are learned from the input data and are utilised to compute the probability corresponding to each symbol during the encoding process [20].

Example

As an example the following text is taken into consideration: `"TTTTAAGAAACCCACTA"`, which is the digital footprint of a user.

- (i) Initialisation stage: we suppose that the dictionary contains the first three characters.
- (ii) Matching stage: the algorithm searches for the longest match between the current position and the dictionary entries. For example, in the

sequence TTTTAAGAAACCCACTA, the longest match at the beginning would be "TTT" with a length of 3 and a distance of 0.

- (iii) Encoding stage: the algorithm encodes the match length, distance, and the next symbol, using probability models learned from the input data. In the example, the match length of 3 would be encoded as $encoded_{ML} = encode(3, |, p_{ML})$, where $p_{ML}(3)$ represents the probability of a match length of 3. The distance of 0 would be encoded as $encoded_{DIST} = encode(0, |, p_{DIST})$, where $p_{DIST}(0)$ represents the probability for a distance of 0. Finally, the next symbol "A" would be encoded as $encoded_{SYM} = encode(\texttt{A}, |, p_{SYM})$, where $p_{SYM}(\texttt{A})$ corresponds to the probability for the symbol "A":

$$\begin{aligned} encoded_{ML} &= encode(3 \,|\, p_{ML}) \\ encoded_{DIST} &= encode(0 \,|\, p_{DIST}) \\ encoded_{SYM} &= encode(\texttt{A} \,|\, p_{SYM}) \end{aligned}$$

- The dictionary is updated with the newly encoded sequence.
  Steps 2-4 are repeated until all input data is compressed.

`bzip2 Algorithm`

This algorithm [21] is based on a combination of Burrows-Wheeler transform (BWT) [22], Move-to-Front (MTF) transform, Run-Length Encoding (RLE) [23], and Huffman coding. The method can be described as follows [21], [22], [23]:

- Burrows-Wheeler Transform (BWT): the input data is first transformed using the BWT, which reorders the input characters so that similar characters are grouped together. The BWT is defined as follows:

$$BWT_i = T(SA_i - 1) \tag{1}$$

  where T symbolises the input data, SA represents the suffix array of T, and i denotes an index. The BWT generates a new string, which is a permutation of the original input data, but with similar characters grouped together.
- Move-to-Front (MTF) Transform: The BWT output is then modified utilising the MTF Transform, which encodes the BWT output by replacing each character in the string with its index in a list of symbols. The MTF Transform is defined as follows: $MTF_i = j$, where j symbolises the position of the character T_i in a list of symbols, and T_i represents the $i - th$ character in the BWT output.
- Run-Length Encoding (RLE): the MTF output is then encoded using RLE encoding, which compresses sequences of repeated symbols in the

MTF output by replacing them with a count and a symbol. For example, if there are 15 consecutive zeros in the MTF output, RLE replaces them with the symbol '0' and the count '15'.

- Huffman Coding: finally, the RLE output is compressed using Huffman coding (see corresponding section)

Example

As an example, the following text: `"TTTTAAGAAACCCACTA"` is considered, which is the digital footprint of a user

After applying the BWT transform, the resulting output is "ATGCAATTTCCCAGAAA". The MTF encoding step transforms the output to "4202012331330100", and the RLE step reduces it to "4212221312110100". Finally, Huffman coding is applied to the RLE output to produce the compressed output, which is significantly smaller than the original input data.

`smaz algorithm`

This algorithm consists of three steps, which can be described as follows [24], [25]:

- (i) Building the dictionary: the dictionary is built by assigning shorter codes to the most frequently occurring substrings.
- (ii) Encoding the input: the input data is encoded by replacing each substring in the input with its corresponding code from the dictionary. The encoded data is stored as a sequence of codes.
- (iii) Decoding the input: the encoded data is decoded by replacing each code in the encoded data with its corresponding substring from the dictionary [25].

Example

As an example the following text is considered `"TTTTAAGAAACCCACTA"`, which is the digital footprint of a user.

The algorithm first builds a dictionary of common substrings in the input string, such as "TA", "AA", "CC", "TTT", etc. These substrings are allocated shorter codes, such as "00", "01", "10", "11", respectively. The input string is then encoded by replacing each substring with its corresponding code, resulting in the encoded string "11 00 01 01 10 00 10 00 10 10 01 00". The encoded string can be decoded by replacing each code with its corresponding substring from the dictionary, resulting in the original decoded string.

S2.2 Building the model

`Generalised Linear Model`:

As is explained in [26], [27] this model considers the output Y_i and the explanatory variables X_i, (X_{i1}, X_{is}) for i = 1, ..., s, in addition to include both a random and a systematic component, as well as a link function. For more information see [26] and [27].

`Support Vector Machine Model`:

As is explained in [28], [29], this model separates a set of examples of $(x_{i1}, \ldots, x_{id}, y_i)$ for i = 1, ..., s training $d - dimensional$ vectors. The values of the explanatory variables correspond to x_i, (x_{i1}, x_{id}) and y_i is the known target. For more information see [28], [29].

`Random Forest Model`:

This algorithm taken into consideration a set of examples $\{x_i, y_i\}$ (SM), in which $x_i = (xi1, ..., xid)$ for i = 1, . . . , s and applies a binary recursive partitions in order to fit a tree. A detailed description of this algorithm can be found in [30].

S2.3 Activity patterns

In order to obtain the activity patterns in humans and bots, the clustering analysis was applied considering the Silhouette [31], [32] and Dunn indexes [33], which are described below:

`Silhouette index`

For each element m in the cluster clu, the $SilW$ parameter is calculated as [31], [32]:

$$SilW_m = \frac{b_m - a_m}{maximum(b_m, a_m)} \tag{2}$$

$$a_m = \frac{\sum_{j \in C_{clu} \quad j \neq m} D_{mj}}{n_{clu} - 1} \tag{3}$$

$$b_m = min_{h \neq clu} \frac{\sum_{j \in C_h}}{n_h} \tag{4}$$

Where

n_{clu} and n_h represent the number of elements in the clu^{th} and h^{th} clusters. If m is the only cluster element [32], $SilW_m$ takes a value equal 0.

The Silhouette coefficient, which takes values in $[-1, 1]$, can be defined as

[32]:

$$SilC = \frac{\sum_{m=1}^{n} SilW_m}{n} \quad (5)$$

The higher the value of SilC, the better the allocation of elements in each cluster [32].

`Dunn index`

The Dunn index is defined as [33]:

$$DunnI = minimum_{1 \leq i < j \leq q} \frac{d(C_i, C_j)}{maximum_{1 \leq j < q} diam(C_k)} \quad (6)$$

Where [33]:

$d(C_i, C_j)$ symbolises a dissimilarity function between C_i and C_j, which is defined as [33]:

$$d(C_i, C_j) = minimum_{x \in C_i, y \in C_j} d(x, y) \quad (7)$$

and $Diameter(C)$ represents the diameter of a cluster C, which is defined as [33]:

$$Diameter(C) = maximum_{x,y \in C} d(x, y) \quad (8)$$

S3 Results

S3.1 Compression algorithms

As we previously explained, in order to obtain the explanatory variables of the model, several compression methods were applied. In particular, the text that describes the types of messages generated by each user (digital footprint) was compressed. For each method, Figure S7 shows the obtained proportion of compression (raw text divided by compress text) as a function of the raw text corresponding to the aforementioned footprint. It can be observed that `gzip` [34], `bzip2` [35], `lzma` [20], and `zlib` [15] algorithms exhibited similar results. In contrast, the `smaz` [25] method showed the highest compression proportion, which increased as the length of the raw text increased. Because of this, `smaz` method is commonly used to compress short strings [36].

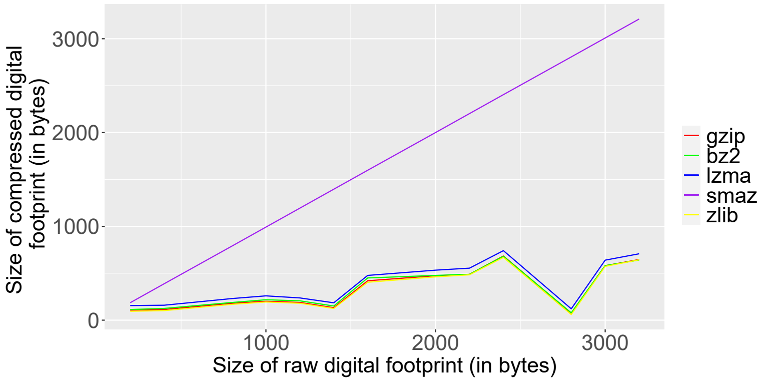

Fig. S7 Comparison of compression algorithms. Ratio of compression is calculated as: [size of raw digital footprint (in bytes)/size of compressed digital footprint (in bytes)]. In the plot the following aspects must be noted (1) Raw footprints are first sorted from lowest to highest and then classified in intervals of 200 elements $[xoi, xei]$ $\forall i = 1 \ldots \mathrm{NumInterval}$, where xoi, xei are the initial and final ends of the i interval, and $NoIntervals$ denotes the number of intervals (ii) As a representation of each interval i, a tuple (xei, $tcri$) is considered, where $tcri$ represents the average value of the compression ratio in the i interval.

Table S1 For each compression algorithm description of the linear fit corresponding to the compression ratio(y) as a function of size of the raw footprint(x) ($y = \beta_0 x + \beta_1 + \epsilon$), ϵ is the error ($E(\epsilon) = 0$, $V(\epsilon) = \sigma^2$). Adjusted R-squared (ARS). Multiple R-squared (MRS) Only the size of raw digital footprint were considered. Correlation Coefficient (CC) is also depicts

Compression algorithm	β_0	β_1	MRS	ARS	CC
`bz2`	0.01071	0.455508	0.81000	0.80990	0.92145
`gzip`	0.01148	1.57778	0.80060	0.80050	0.91188
`lzma`	7.16200e-03	7.08200e-01	0.85870	0.85860	0.93940
`smaz`	-4.63200e-05	1.12200e+00	0.39380	0.39340	-0.9719434
`zlib`	0.01333	2.22245	0.7613	0.7611	0.8923908

Table S2 For each compression algorithm, raw size of the digital footprint (SDF) that generates the higher number of dots lying above (nop) and below (non) the regression line)

Compression algorithm	SDF	non	SDF	nop
`bz2`	3,265	1,431	3,265	153
`gzip`	3,265	1,438	3,265	146
`lzma`	3,265	1,493	3,265	91
`smaz`	3,265	1,584	3,265;155;170;274;286;314;651,727; 936;963;2231;2885;2,904	3
`bzlib`	3,265	1,431	3,265	153

Table S3 For bots and humans, statistical quartiles (25%, 50%, 75% and 100%) corresponding to the size of the raw digital footprint

Account Typology	25%	50%	75%	100%
`Bots`	2,747.75	3,265	3265	3,324.00
`Humans`	1,649.5	3,221	3,290	3,332

S3.2 Building the model

S3.2.1 `Alternative 1`. Explanatory variables

For `Alternative 1`, optimum GLM, RFM and SVM models, Tables S4 and S5 depict the parameters and performance metrics of the model. For `zlib`, `bzip2`, `gzip`, `smaz` and `lzma` algorithms, Tables S7, S8, S9, S10, and S6 display the confusion matrix for training and validation sets.

Table S4 For `Alternative 1`, optimum parameters corresponding to the Random Forest (RFM) and support vector machine (SVM) models by compression algorithm. `mtry`: it symbolises the number of variables that have been randomly selected to be sampled in each split. `min.node.size`: it represents the minimal node size. `splitrule`: it denotes the used splitting rule. `no. trees` is the number of trees.

		`zlib` algorithm		
		RFM		SVM
`mtry`	`splitrule`	`min.node.size`	`no. trees`	`C`
1	gini	1	500	1.4737
		bzip2 algorithm		
		RFM		SVM
`mtry`	`splitrule`	`min.node.size`	`no. trees`	`C`
1	gini	1	500	1.4737
		gzip algorithm		
		RFM		SVM
`mtry`	`splitrule`	`min.node.size`	`no. trees`	`C`
1	gini	1	150	2
		lzma algorithm		
		RFM		SVM
`mtry`	`splitrule`	`min.node.size`	`no. trees`	`C`
1	gini	1	150	1.4737
		smaz algorithm		
		RFM		SVM
`mtry`	`splitrule`	`min.node.size`	`no. trees`	`C`
2	gini	1	150	1.2105

Table S5 For Alternative 1, generalised linear (*GLM*), random forest (*RFM*) and support vector machine (*SVM*) models, performance metrics obtained in each compression algorithm. TS: Training Stage. VS: Validation Stage. Acc: Accuracy, Sen: Sensitivity, Spe: Specificity, Kap: Kappa.

zlib algorithm														
TS	VS				TS	VS				TS	VS			
GLM					RFM					SVM				
Acc	Acc	Sen	Spe	Kap	Acc	Acc	Sen	Spe	Kap	Acc	Acc	Sen	Spe	Kap
0.8304	0.8329	0.8191	0.9655	0.4444	0.8808	0.8854	0.8821	0.9172	0.5429	0.8134	0.8037	0.7888	0.9476	0.3899
bzip2 algorithm														
GLM					RFM					SVM				
TS	VS				TS	VS				TS	VS			
Acc	Acc	Sen	Spe	Kap	Acc	Acc	Sen	Spe	Kap	Acc	Acc	Sen	Spe	Kap
0.8282	0.8329	0.8204	0.9531	0.4408	0.8825	0.8857	0.8817	0.9241	0.5455	0.8134	0.7956	0.7799	0.9462	0.3769
gzip algorithm														
GLM					RFM					SVM				
TS	VS				TS	VS				TS	VS			
Acc	Acc	Sen	Spe	Kap	Acc	Acc	Sen	Spe	Kap	Acc	Acc	Sen	Spe	Kap
0.8290	0.8334	0.8206	0.9572	0.443	0.8791	0.8876	0.8848	0.9145	0.5477	0.8135	0.8111	0.7978	0.9393	0.3993
lzma algorithm														
GLM					RFM					SVM				
TS	VS				TS	VS				TS	VS			
Acc	Acc	Sen	Spe	Kap	Acc	Acc	Sen	Spe	Kap	Acc	Acc	Sen	Spe	Kap
0.8262	0.8326	0.8193	0.9614	0.4427	0.8759	0.8814	0.8765	0.9283	0.5361	0.8089	0.8019	0.7864	0.9517	0.3882
smaz algorithm														
GLM					RFM					SVM				
TS	VS				TS	VS				TS	VS			
Acc	Acc	Sen	Spe	Kap	Acc	Acc	Sen	Spe	Kap	Acc	Acc	Sen	Spe	Kap
0.3703	0.3818	0.3435	0.7503	0.0252	0.7173	0.7287	0.7270	0.7448	0.2285	0.3146	0.3137	0.26513	0.7821	0.0116

`zlib algorithm`

Table S6 For alternative 1 with zlib algorithm, confusion matrix corresponding to training and validation sets in each used model. AV 0: Actual value "not bot". AV 1: Actual value "bot". PV 0: Prediction value "not bot", PV 1: Prediction value "bot".

	TS						VS					
	GLM		RFM		SVM		GLM		RFM		SVM	
	AV 0	AV 1	AV 0	AV 1	AV 0	AV 1	AV 0	AV 1	AV 0	AV 1	AV 0	AV 1
PV 0	74.10%	0.40%	79.50%	0.80%	72.40%	0.40%	5725	25	6165	60	5513	38
PV 1	16.50%	9.00%	13.00%	6.30%	18.20%	9.00%	1264	700	824	665	1476	687

`bzip2 algorithm`

Table S7 For alternative 1 with bzip2 algorithm, confusion matrix corresponding to training and validation sets in each used model. AV 0: Actual value "not bot". AV 1: Actual value "bot". PV 0: Prediction value "not bot", PV 1: Prediction value "bot".

	TS						VS					
	GLM		RFM		SVM		GLM		RFM		SVM	
	AV 0	AV 1	AV 0	AV 1	AV 0	AV 1	AV 0	AV 1	AV 0	AV 1	AV 0	AV 1
PV 0	73.8%	0.4%	79.6%	0.8%	71.3%	0.4%	5734	34	6112	55	5451	39
PV 1	16.8%	9%	11%	8.6%	19.3%	9%	1255	691	827	670	1538	686

`gzip algorithm`

Table S8 For alternative 1 with gzip algorithm, confusion matrix corresponding to training and validation sets in each used model. AV 0: Actual value "not bot". AV 1: Actual value "bot". PV 0: Prediction value "not bot", PV 1: Prediction value "bot".

	TS						VS					
	GLM		RFM		SVM		GLM		RFM		SVM	
	AV 0	AV 1	AV 0	AV 1	AV 0	AV 1	AV 0	AV 1	AV 0	AV 1	AV 0	AV 1
PV 0	73.90%	0.40%	79.30%	0.80%	71.30%	0.40%	5735	31	6184	62	5576	44
PV 1	73.90%	0.40%	11.30%	8.60%	19.30%	9.00%	1254	694	805	663	1413	681

`lzma algorithm`

Table S9 For alternative 1 with lzma algorithm, confusion matrix corresponding to training and validation sets in each used model. AV 0: Actual value "not bot". AV 1: Actual value "bot". PV 0: Prediction value "not bot", PV 1: Prediction value "bot".

	TS						VS					
	GLM		RFM		SVM		GLM		RFM		SVM	
	AV 0	AV 1	AV 0	AV 1	AV 0	AV 1	AV 0	AV 1	AV 0	AV 1	AV 0	AV 1
PV 0	73.60%	0.40%	78.90%	0.80%	71.90%	0.40%	5726	28	6126	52	5496	35
PV 1	17.00%	9.00%	11.70%	8.60%	18.70%	9.00%	1263	697	863	673	1493	690

`smza algorithm`

Table S10 For alternative 1 with smaz algorithm, confusion matrix corresponding to training and validation sets in each used model. AV 0: Actual value "not bot". AV 1: Actual value "bot". PV 0: Prediction value "not bot", PV 1: Prediction value "bot".

	TS						VS					
	GLM		RFM		SVM		GLM		RFM		SVM	
	AV 0	AV 1	AV 0	AV 1	AV 0	AV 1	AV 0	AV 1	AV 0	AV 1	AV 0	AV 1
PV 0	29.90%	2.20%	64.80%	2.40%	23.90%	1.80%	2401	181	5081	185	1853	158
PV 1	60.70%	7.20%	25.80%	7%	66.70%	7.60%	4588	544	1908	540	5136	567

S3.2.2 `Alternative 3`. Explanatory variables

For `Alternative 3`, GLM, RFM and SVM models, Tables S11 and S12 display optimum parameters and performance metrics of the model. Tables S15. S13, S16, S17, and S14 display for training and validation sets the confusion matrix in each compression algorithm.

Table S11 For `Alternative 3`, optimum parameters corresponding to the random forest (RFM) and support vector machine (SVM) models by compression algorithm. `mtry`: it symbolises the number of variables that have been randomly selected to be sampled in each split. `min.node.size`: it represents the minimal node size. `splitrule`: it denotes the used splitting rule. `no. trees` is the number of trees.

		zlib algorithm		
		RFM		SVM
mtry	splitrule	min.node.size	trees	C
1	gini	1	300	1.2105
		bzip2 algorithm		
		RFM		SVM
mtry	splitrule	min.node.size	trees	C
2	gini	1	300	1.2105
		gzip algorithm		
		RFM		SVM
mtry	splitrule	min.node.size	trees	C
2	gini	1	300	1
		lzma algorithm		
		RFM		SVM
mtry	splitrule	min.node.size	trees	C
2	gini	1	300	1.2105
		smaz algorithm		
		RFM		SVM
mtry	splitrule	min.node.size	trees	C
2	gini	1	300	1.0526

Table S12 For Alternative 3, generalised linear (*GLM*), random forest (*RFM*) and support vector machine (*SVM*) models, performance metrics obtained in each compression algorithm. TS: Training Stage. VS: Validation Stage. Acc: Accuracy, Sen: Sensitivity, Spe: Specificity, Kap: Kappa.

zlib algorithm														
TS	VS				TS	VS				TS	VS			
Acc	Acc	Sen	Spe	Kap	Acc	Acc	Sen	Spe	Kap	Acc	Acc	Sen	Spe	Kap
GLM					RFM					SVM				
0.8304	0.8329	0.8191	0.9655	0.4444	0.8779	0.8855	0.8825	0.9145	0.5425	0.8408	0.8290	0.8152	0.9638	0.4351
bzip2 algorithm														
TS	VS				TS	VS				TS	VS			
Acc	Acc	Sen	Spe	Kap	Acc	Acc	Sen	Spe	Kap	Acc	Acc	Sen	Spe	Kap
GLM					RFM					SVM				
0.8426	0.8395	0.8296	0.9378	0.4443	0.896	0.9008	0.8978	0.9921	0.5853	0.836	0.8254	0.8112	0.9638	0.4287
gzip algorithm														
TS	VS				TS	VS				TS	VS			
GLM					RFM					SVM				
Acc	Acc	Sen	Spe	Kap	Acc	Acc	Sen	Spe	Kap	Acc	Acc	Sen	Spe	Kap
0.8366	0.8381	0.8257	0.9615	0.4470	0.8971	0.9000	0.8966	0.9331	0.5839	0.8388	0.8375	0.8252	0.9568	0.4485
lzma algorithm														
TS	VS				TS	VS				TS	VS			
Acc	Acc	Sen	Spe	Kap	Acc	Acc	Sen	Spe	Kap	Acc	Acc	Sen	Spe	Kap
GLM					RFM					SVM				
0.8388	0.8369	0.8250	0.9544	0.4428	0.8972	0.9006	0.8968	0.9373	0.5864	0.835	0.8251	0.8107	0.9652	0.4286
smaz algorithm														
TS	VS				TS	VS				TS	VS			
Acc	Acc	Sen	Spe	Kap	Acc	Acc	Sen	Spe	Kap	Acc	Acc	Sen	Spe	Kap
GLM					RFM					SVM				
0.7941	0.7993	0.7842	0.9477	0.3788	0.8961	0.8989	0.8949	0.9373	0.5864	0.8001	0.7898	0.7721	0.9624	0.3714

`gzip algorithm`

Table S13 For alternative 3 with gzip algorithm, confusion matrix corresponding to training and validation sets in each used model. AV 0: Actual value "not bot". AV 1: Actual value "bot". PV 0: Prediction value "not bot", PV 1: Prediction value "bot".

	TS						VS					
	GLM		RFM		SVM		GLM		RFM		SVM	
	AV 0	AV 1	AV 0	AV 1	AV 0	AV 1	AV 0	AV 1	AV 0	AV 1	AV 0	AV 1
PV 0	74.90%	0.40%	81.00%	0.60%	75.10%	0.40%	5754	27	6263	48	5754	31
PV 1	15.90%	8.80%	9.60%	8.70%	15.70%	8.80%	1215	675	722	670	1221	687

`zlib algorithm`

Table S14 For alternative 3 with zlib algorithm, confusion matrix corresponding to training and validation sets in each used model. AV 0: Actual value "not bot". AV 1: Actual value "bot". PV 0: Prediction value "not bot", PV 1: Prediction value "bot".

	TS						VS					
	GLM		RFM		SVM		GLM		RFM		SVM	
	AV 0	AV 1	AV 0	AV 1	AV 0	AV 1	AV 0	AV 1	AV 0	AV 1	AV 0	AV 1
PV 0	74.10%	0.40%	79.10%	0.70%	75.30%	0.4%	5725	25	6168	62	5694	26
PV 1	16.50%	9%	11.50%	8.70%	15.50%	8.80%	1264	700	821	663	1291	692

`bzip2 algorithm`

Table S15 For alternative 3 with bzip2 algorithm, confusion matrix corresponding to training and validation sets in each used model. AV 0: Actual value "not bot". AV 1: Actual value "bot". PV 0: Prediction value "not bot", PV 1: Prediction value "bot".

	TS						VS					
	GLM		RFM		SVM		GLM		RFM		SVM	
	AV 0	AV 1	AV 0	AV 1	AV 0	AV 1	AV 0	AV 1	AV 0	AV 1	AV 0	AV 1
PV 0	75.50%	0.40%	80.90%	0.60%	74.80%	0.40%	5782	44	6271	60	5666	26
PV 1	15.30%	9.00%	9.80%	8.70%	16.00%	8.80%	1188	73	714	668	1319	692

`lzma algorithm`

Table S16 For alternative 3 with lzma algorithm, confusion matrix corresponding to training and validation sets in each used model. AV 0: Actual value "not bot". AV 1: Actual value "bot". PV 0: Prediction value "not bot", PV 1: Prediction value "bot".

	TS						VS					
	GLM		RFM		SVM		GLM		RFM		SVM	
	AV 0	AV 1	AV 0	AV 1	AV 0	AV 1	AV 0	AV 1	AV 0	AV 1	AV 0	AV 1
PV 0	75.20%	0.50%	81.00%	0.60%	75.00%	0.40%	5748	32	6254	45	5663	25
PV 1	15.6%	8.70%	9.60%	8.70%	15.80%	8.80%	1219	670	721	673	1322	693

`smaz algorithm`

Table S17 For alternative 1 with smaz algorithm, confusion matrix corresponding to training and validation sets in each used model. AV 0: Actual value "not bot". AV 1: Actual value "bot". PV 0: Prediction value "not bot", PV 1: Prediction value "bot".

	TS						VS					
	GLM		RFM		SVM		GLM		RFM		SVM	
	AV 0	AV 1	AV 0	AV 1	AV 0	AV 1	AV 0	AV 1	AV 0	AV 1	AV 0	AV 1
PV 0	70.60%	0.40%	80.90%	0.60%	71.10%	0.30%	5467	37	6251	45	5393	27
PV 1	20.20%	8.70%	9.80%	8.70%	19.70%	8.90%	1504	670	734	673	1592	691

S3.3 Account typologies

`Type A interactions`

For type A interactions and human users, Figure S8 displays the box plots corresponding to *plag* and $p - value$ associated with the centroids in each cluster, which were obtained as result of the 100 carried out experiments. Figure S11 depicts similar results for mean, standard deviation, mode, minimum and maximum values. Figure S9, Figure S10, Figure S12 and Figure S13 show the histograms corresponding to each parameter on the basis of which the clusters 1 and 2 were constructed.

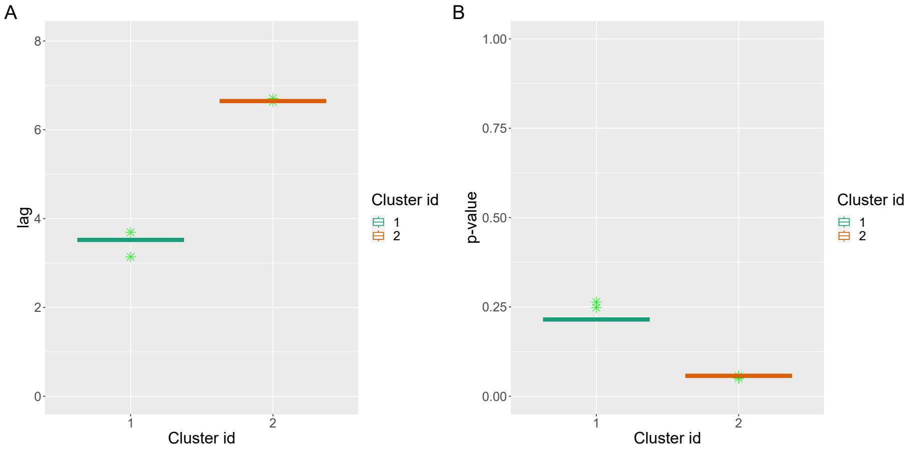

Fig. S8 For type A interactions, for human users, boxplots corresponding to *plag* and *p-value* associated with the centroids in each cluster. These clusters were obtained as a result of the execution of the 100 experiments. `lag` (mean = 5.08 (± 1.57), median = 5.16), *p*−*value* (mean = 0.14 (± 0.08), median = 0.14). Next to the mean, standard deviation is indicated in brackets

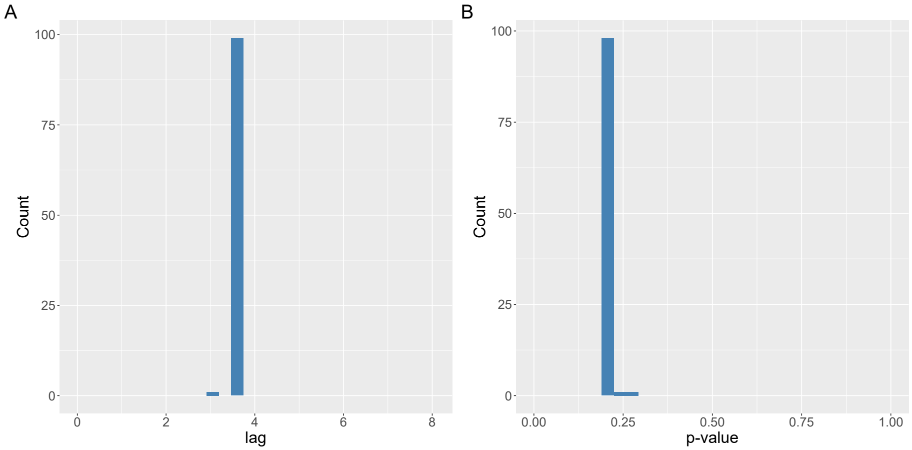

Fig. S9 For type A interactions, for human users, histogram corresponds to *plag* and *p-value* associated with the centroids in cluster 1, which were obtained in the 100 experiments.

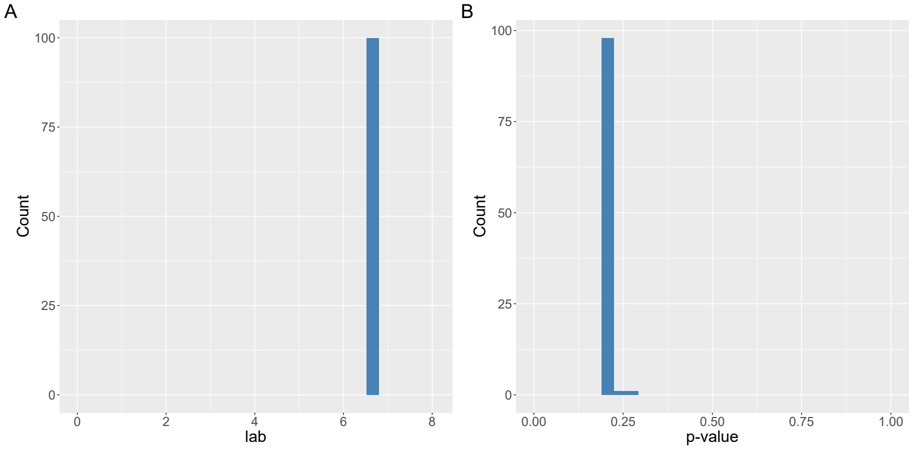

Fig. S10 For type A interactions, for human users, histogram corresponds to *plag* and *p-value* associated with the centroids in cluster 2, which were obtained in the 100 experiments.

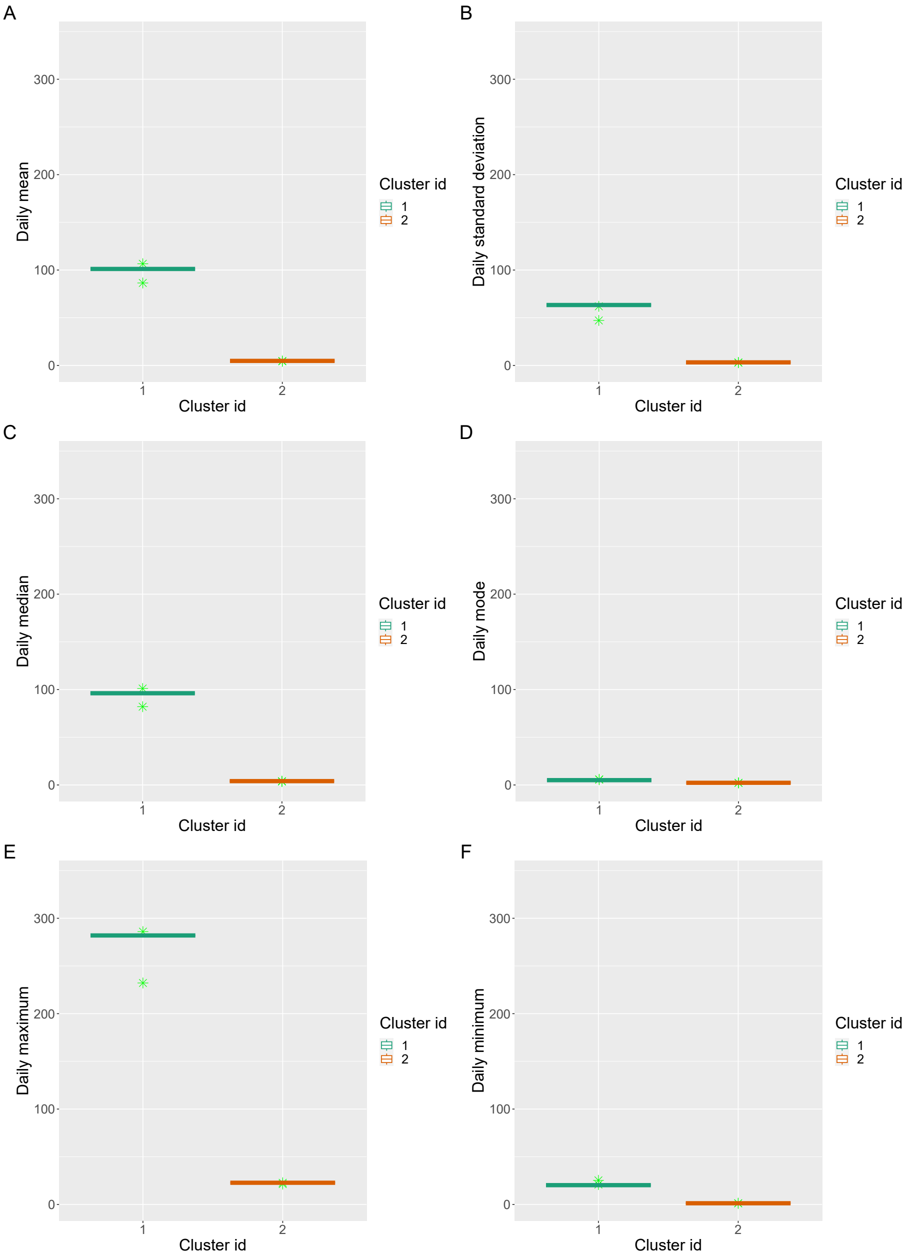

Fig. S11 For type A interactions, for human users, box plots correspond to *mean*, *sd*, *mode*, *median*, *maximum* and *minimum* associated with the centroids in each cluster, which were obtained in the 100 experiments. `average` (mean = 52.91 (± 48.28), median = 45.62), `median` (mean = 50.03 (± 46.14), median = 43.04), `mode` (mean = 3.66 (± 1.4), median = 3.65), `maximum` (mean = 152.14 (± 129.76), median = 127.47), `minimum` (mean = 10.78 (± 9.55), median = 10.75)

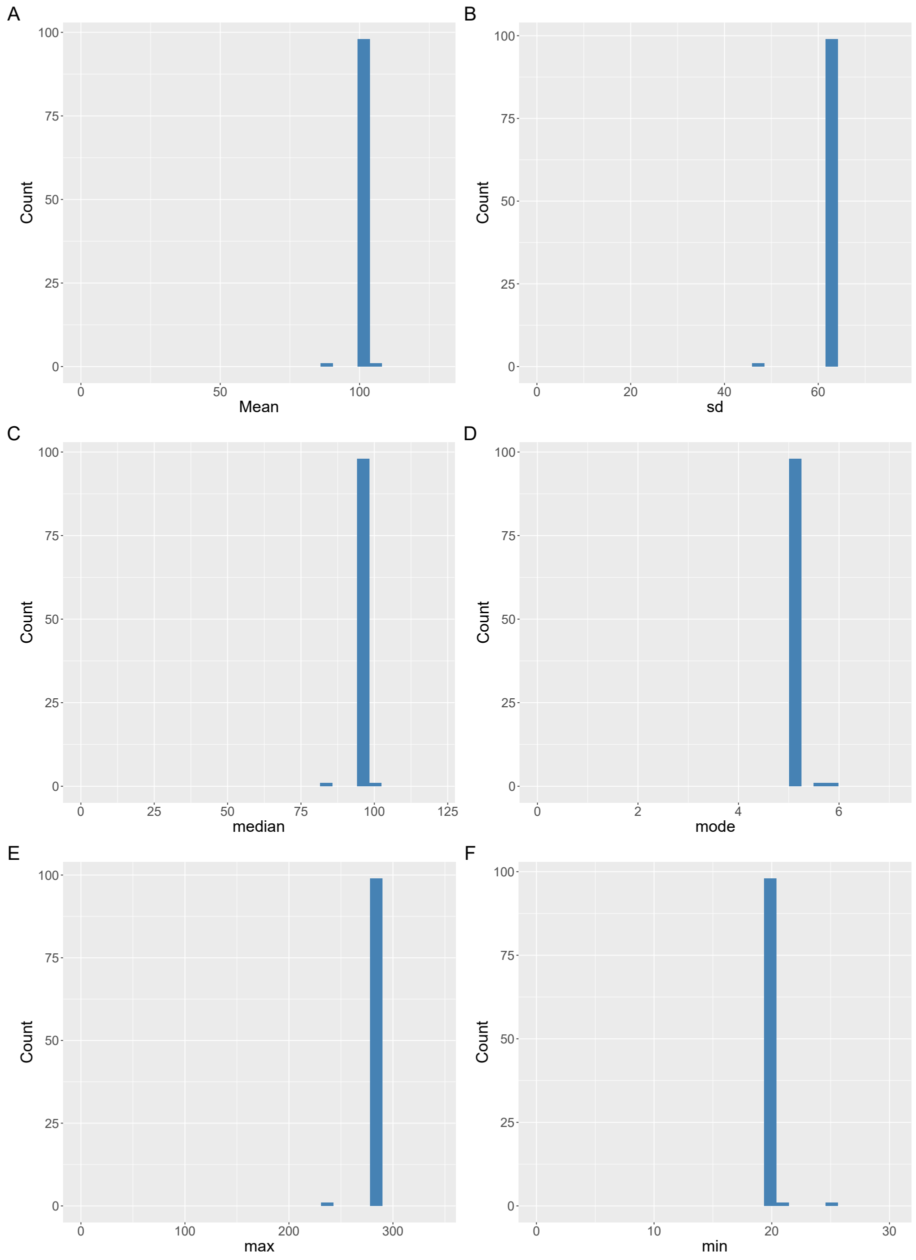

Fig. S12 For type A interactions, for human users, histograms correspond to *mean*, *sd*, *mode*, *median*, *maximum* and *minimum* values associated with the centroids in cluster 1, which were obtained in the 100 experiments.

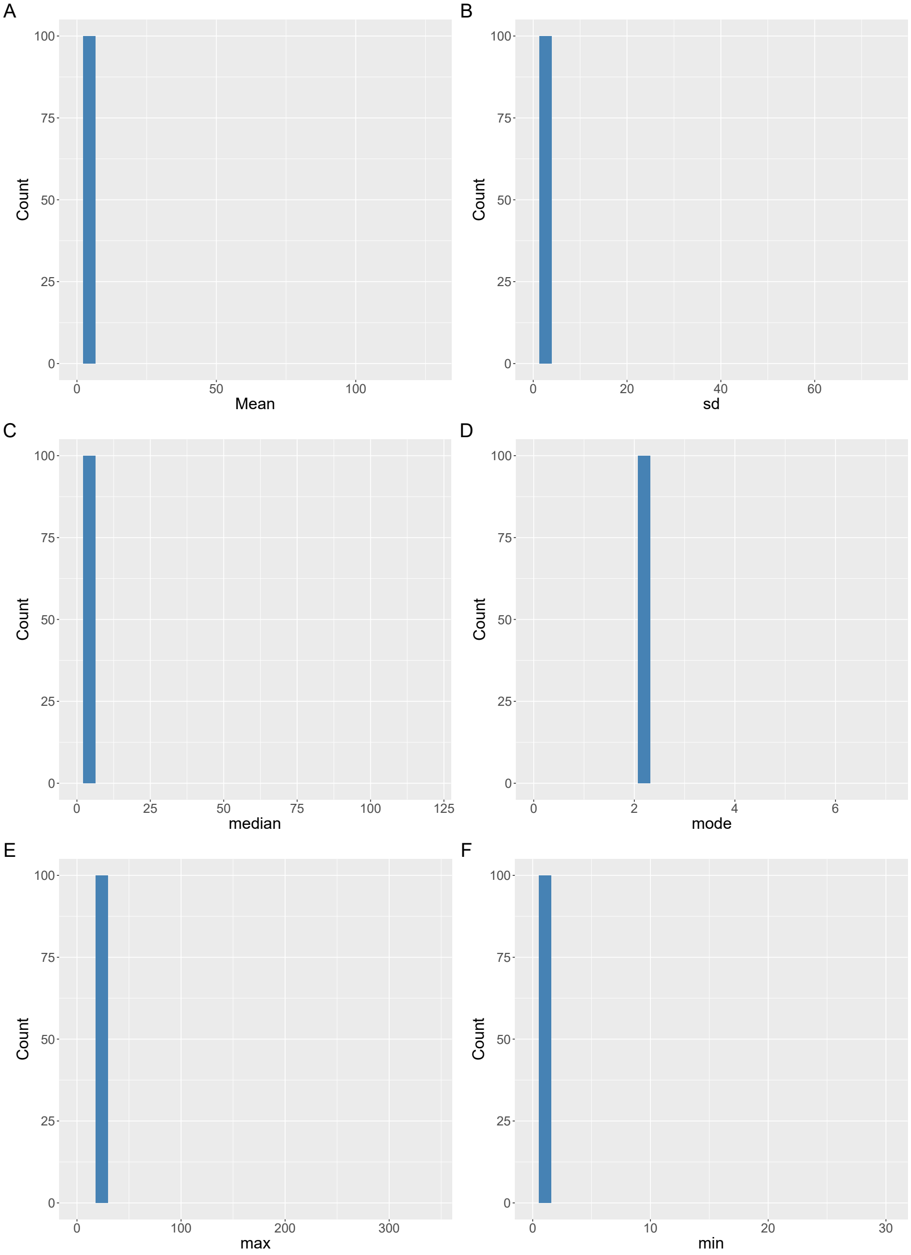

Fig. S13 For type A interactions, for human users, histograms correspond to *mean*, *sd*, *mode*, *median*, *maximum* and *minimum* values associated with the centroids in cluster 2, which were obtained in the 100 experiments.

Type T interactions

For type T interactions and human users, Figure S14 displays the box plots corresponding to *plag* and $p - value$ associated with the centroids in each cluster, which were obtained as result of the 100 carried out experiments. Figure S17 depicts similar results for mean, standard deviation, mode, minimum and maximum values. Figure S15, S16, S18 and S19 show the histograms corresponding to each parameter on the basis of which the clusters 1 and 2 were constructed.

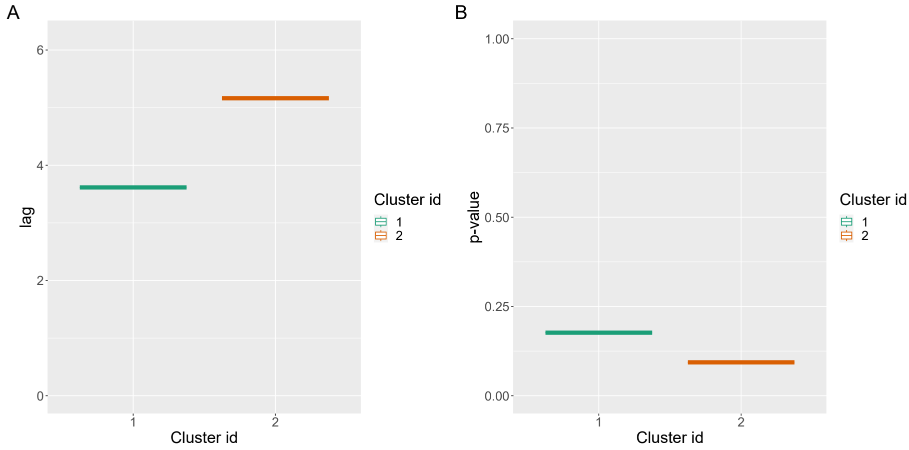

Fig. S14 For type T interactions, for human users, boxplots corresponding to *plag* and *p-value* associated with the centroids in each cluster. These clusters were obtained as a result of the execution of the 100 experiments. `lag` (mean=4.39 ($\pm$ 0.78), median = 4.39), p-*value* (mean = 0.14 ($\pm$ 0.04) Median = 0.14), Next to the mean, standard deviation is indicated in brackets

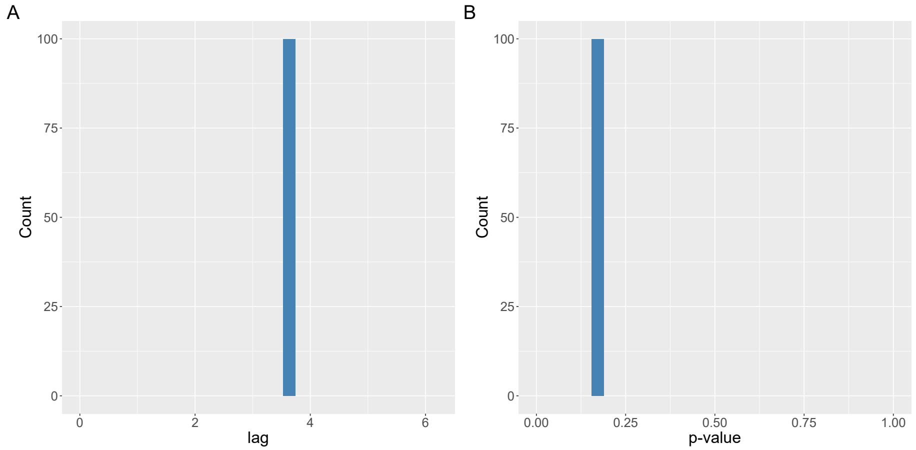

Fig. S15 For type T interactions, for human users, histograms correspond to *plag* and *p-value* associated with the centroids in cluster 1, which were obtained in the 100 experiments.

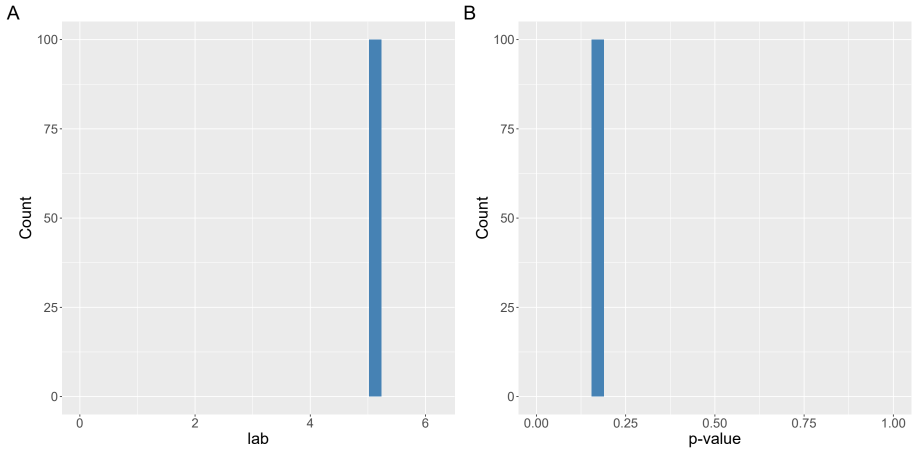

Fig. S16 For type T interactions, for human users, histogram corresponds to *plag* and *p-value* associated with the centroids in cluster 2, which were obtained in the 100 experiments.

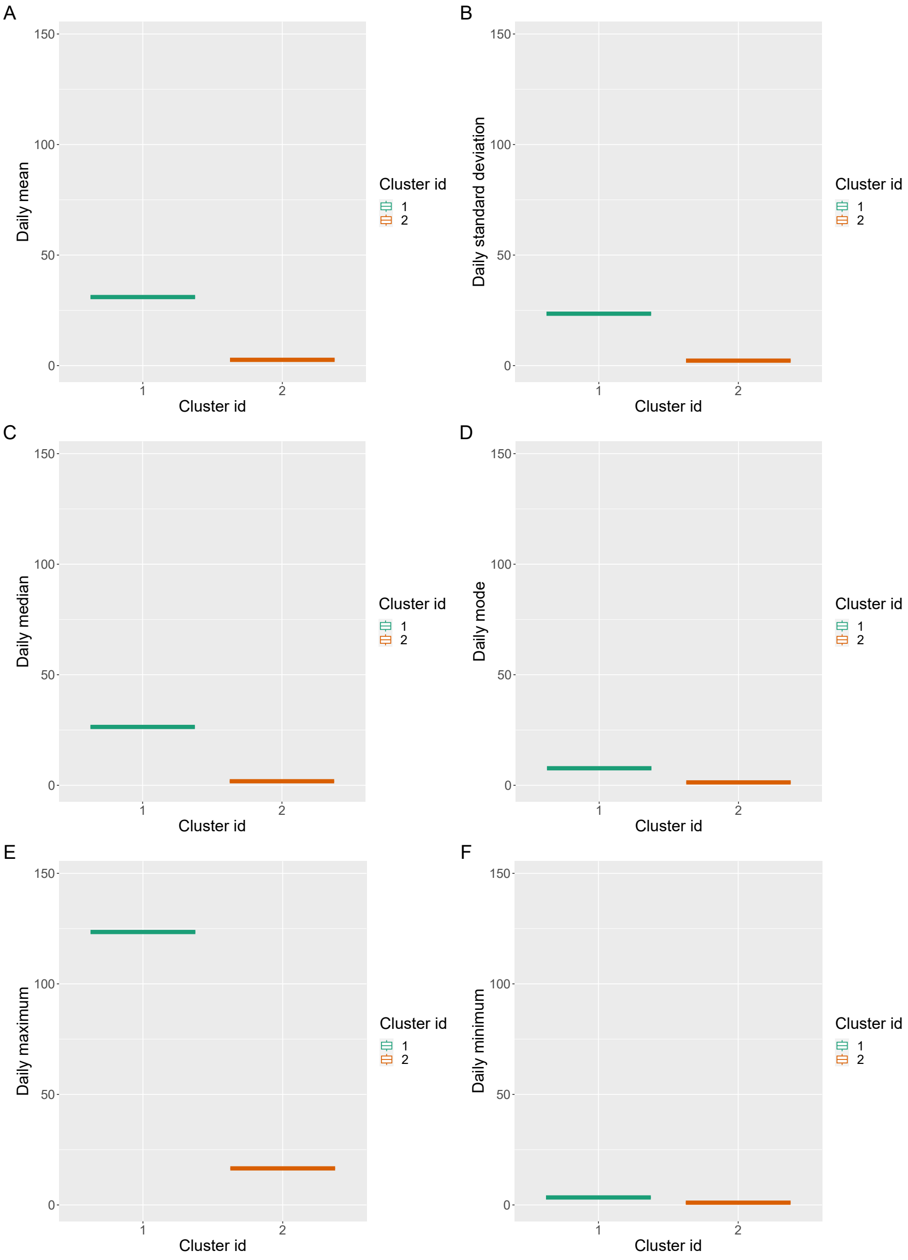

Fig. S17 For type T interactions, for human users, boxplots corresponds to *mean*, *sd*, *mode*, *median*, *maximum* and *minimum* associated with the centroids in each cluster, which were obtained in the 100 experiments. `average` (mean = 16.81 (± 14.23) median = 16.81), `median` (mean = 14.1 (± 12.31) median = 14.1), `mode` (mean = 4.49 (± 3.21) median = 4.49), `maximum` (mean = 70 (± 53.6) median = 70), `minimum` (mean = 2.21 (± 1.18) median = 2.21)

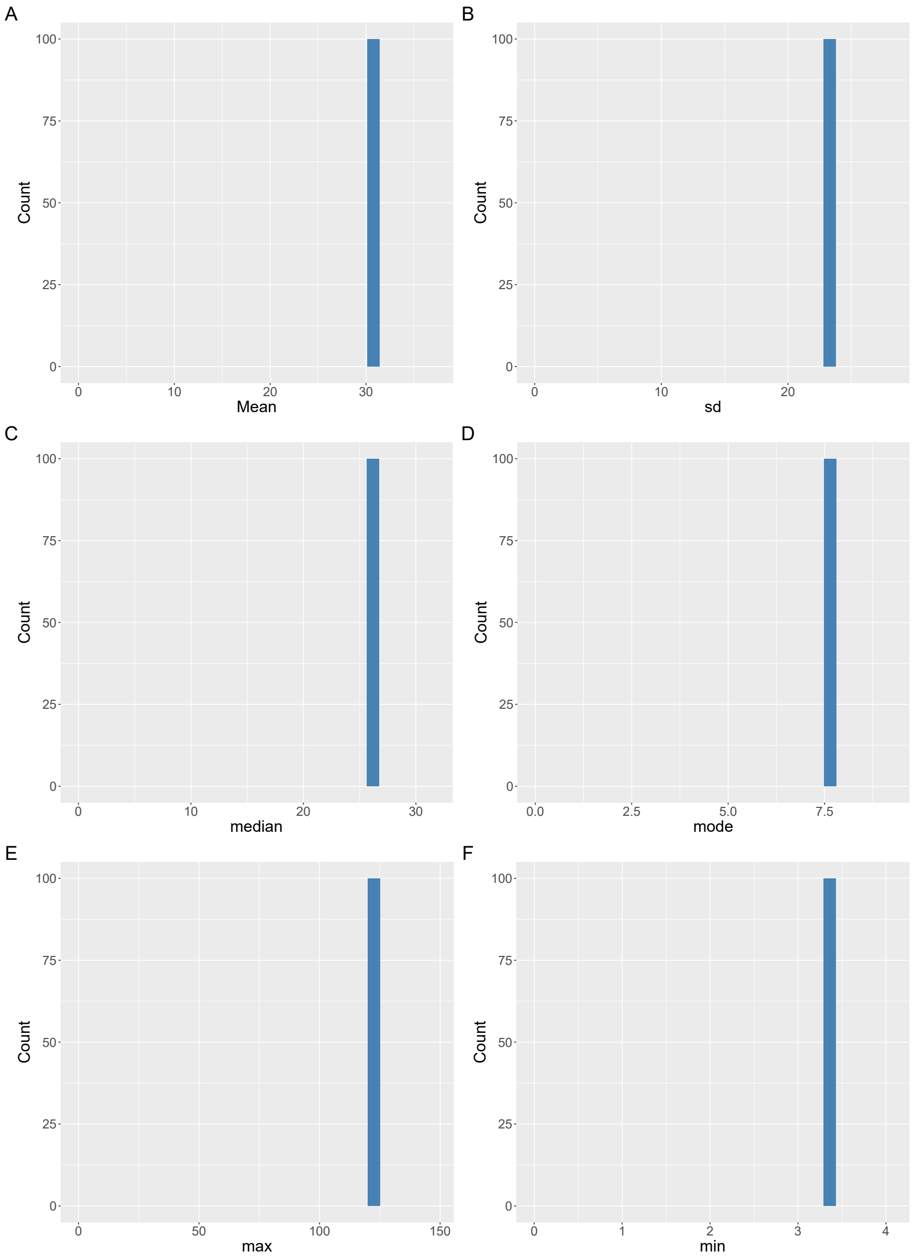

Fig. S18 For type T interactions, for human users, histograms correspond to *mean*, *sd*, *mode*, *median*, *maximum* and *minimum* values associated with the centroids in cluster 1, which were obtained in the 100 experiments.

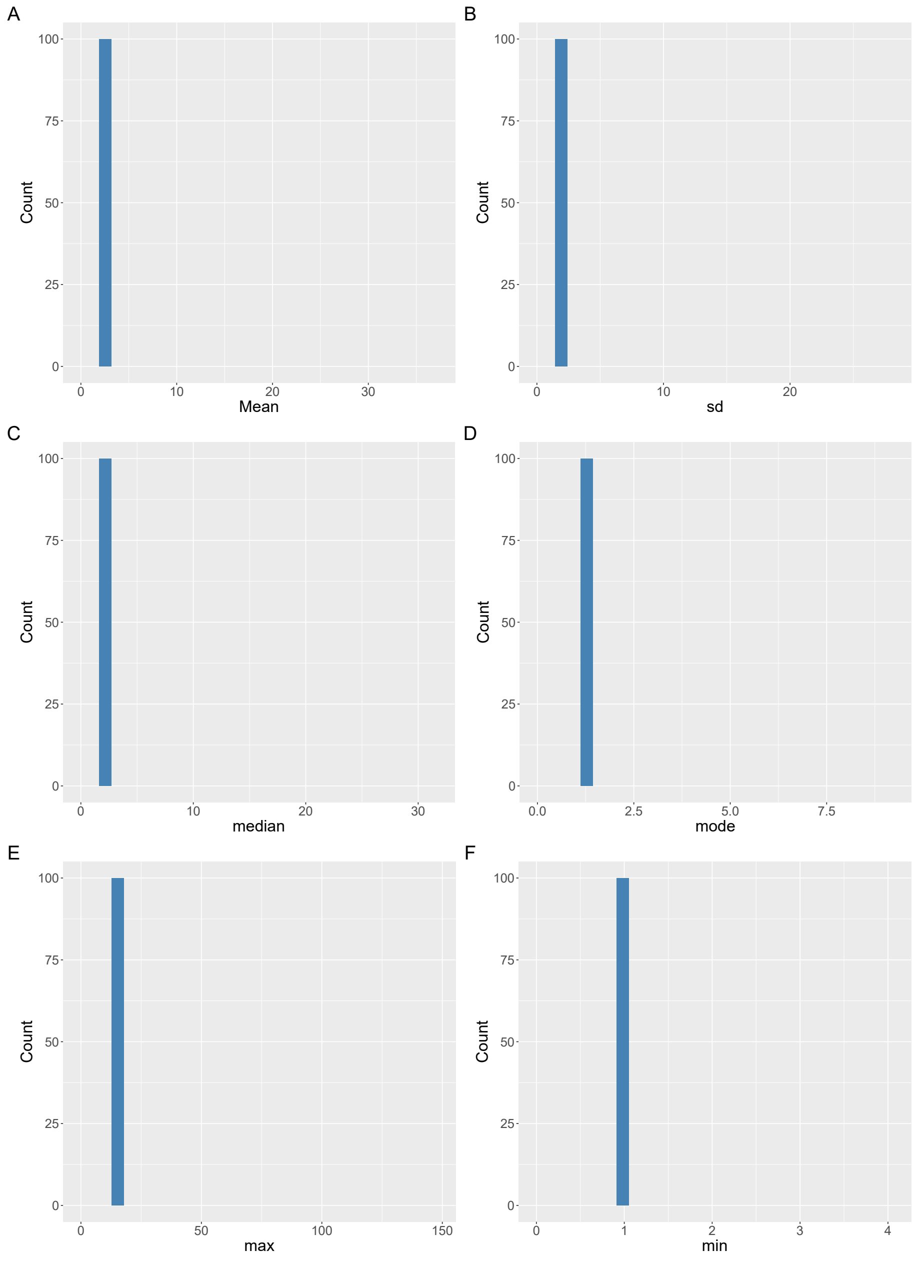

Fig. S19 For type T interactions, for human users, histograms correspond to *mean*, *sd*, *mode*, *median*, *maximum* and *minimum* values associated with the centroids in cluster 2, which were obtained in the 100 experiments.

S3.4 Sentiment Analysis

For the selected 38,615 users, for human and bots users, Figure S20 displays the evolution of sentiment from 2006 to 2022 by cluster. For all users who posted about sustainability (using the chosen keywords), Figure S21 displays the evolution of sentiment from January to December 2022. It can be seen that the sentiment is mostly positive.

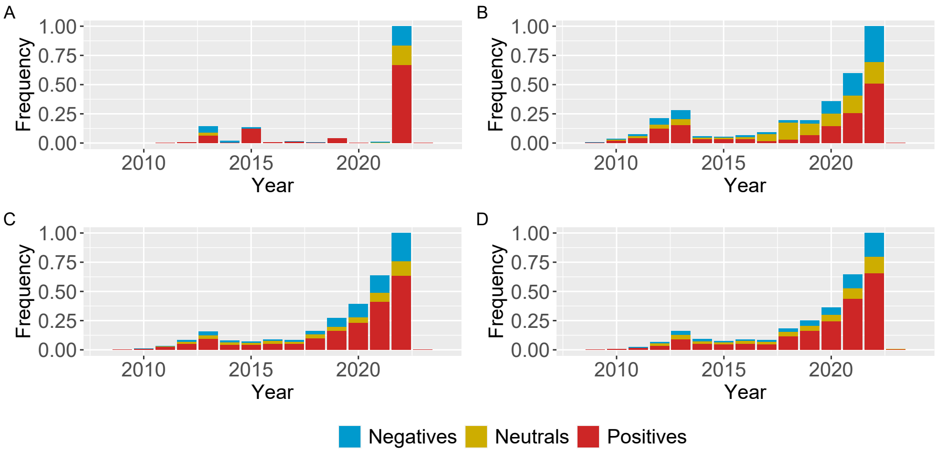

Fig. S20 Evolution of sentiment polarity by clusters. A: Bots Cluster 1, B: Bots Cluster 2, C: Humans Cluster 1, D: Humans Cluster 2.

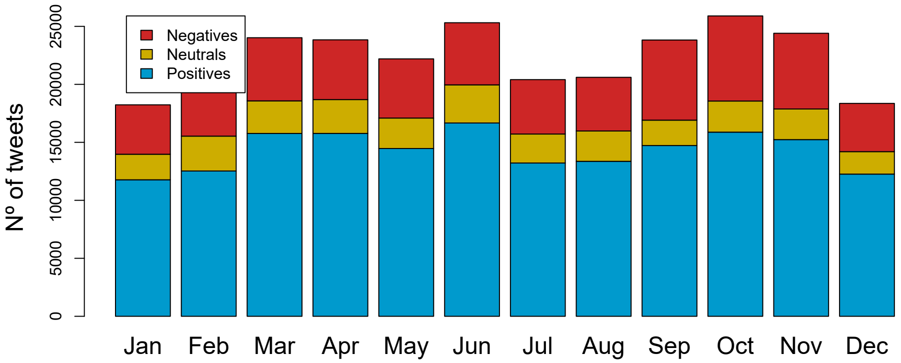

Fig. S21 Evolution of sentiment polarity in 2022.

Table S18 Number of days in which the average sentiment reached extreme values during the first 4 months of 2022 (120 days).

**Average daily sentiment**	**Bots Number of days**	**Humans Number of days**
< 0.05	19/120	2/120
> 0.1	23/120	10/120

S3.5 Keywords Analysis

Table S19 Cosine distance between the top 20 words of each cluster.

	**All**	**Bots 1**	**Bots 2**	**Humans 1**	**Humans 2**
**All**	1	0.81	0.92	0.89	0.93
**Bots 1**	0.81	1	0.91	0.82	0.79
**Bots 2**	0.92	0.91	1	0.95	0.89
**Humans 1**	0.89	0.82	0.95	1	0.88
**Humans 2**	0.93	0.79	0.89	0.88	1

Table S20 Top keywords by cluster (Word count method).

**All**	**Bots Cluster 1**	**Bots Cluster 2**	**Humans Cluster 1**	**Humans Cluster 2**
**energy**	raleigh	air	**energy**	**energy**
renewable	carolina	**energy**	renewable	renewable
air	san	**pollution**	**pollution**	**pollution**
**pollution**	**pollution**	renewable	air	air
sustainable	francisco	quality	sustainable	sustainable
new	**energy**	health	insight	new
food	44401	forecast	successful	food
health	579171968	advice	real	city
quality	177	good	lungs	power
power	raleighnorth	consider	new	health
city	157	low	industry	clean
forecast	twitterfeed	facebook	make	help
facebook	air	moderate	production	solar
solar	renewable	index	key	water
industry	20130506	central	siemens	twitter
green	now	london	expert	green
water	3323999	particle	largescale	industry
project	3154	aqi	plan	project
make	60452453	micron	give	climate
help	around	update	tea	make

References

[1] Davis, C., Varol, O., Ferrara, E., Flammini, A., Menczer, F.: Botornot: A system to evaluate the veracity of online identities. In: Proceedings of the 25th International Conference Companion on World Wide Web, pp. 273–274 (2016). ACM

[2] Summers, E.: Twarc2. https://github.com/DocNow/twarc. Version 2.4.0 (2021)

[3] Mouronte-López, M.L., Subirán, M.: What do twitter users think about climate change? characterization of twitter interactions considering geographical, gender, and account typologies perspectives. Weather, Climate, and Society **14**, 1039–1064 (2022). https://doi.org/10.1175/WCAS-D-21-0163.1

[4] Mouronte, M.L., Savall Ceres, J., Mora Columbrans, A.: Analysing the sentiments about the education system trough twitter. Education and Information Technologies **28**, 1–30 (2023). https://doi.org/10.1007/s10639-022-11493-8

[5] Mouronte-López, M.L., Subirán, M.: Modeling the interaction networks about the climate change on twitter: A characterization of its network structure. Complexity **2022**, 1–20 (2022). https://doi.org/10.1155/2022/8924468

[6] Congosto, M.L., Basanta-Val, P., Sanchez-Fernandez, L.: T-hoarder: A framework to process twitter data streams. Journal of Network and Computer Applications **83**, 28–39 (2017). https://doi.org/10.1016/j.jnca.2017.01.029

[7] Huffman, D.A.: A method for the construction of minimum-redundancy codes. Proceedings of the IRE **40**(9), 1098–1101 (1952). https://doi.org/10.1109/JRPROC.1952.273898

[8] Mukti, Z., Akter, S.: A new approach of a memory efficient huffman tree representation technique, pp. 731–736 (2012). https://doi.org/10.1109/ICIEV.2012.6317482

[9] Moffat, A.: Huffman coding. ACM Computing Surveys **52**, 1–35 (2019). https://doi.org/10.1145/3342555

[10] Online, S.: Introduction to Huffman Coding. https://www.shiksha.com/online-courses/articles/introduction-to-huffman-coding/ (2023)

[11] of Science, T.H.K.U., Technology: Lecture 17: Huffman Coding CLRS- 16.3. https://home.cse.ust.hk/faculty/golin/COMP271Sp03/Notes/MyL17.pdf (w.d.)

[12] Rigler, S., Bishop, W., Kennings, A.: Fpga-based lossless data compression using huffman and lz77 algorithms, pp. 1235–1238 (2007). https://doi.org/10.1109/CCECE.2007.315

[13] Afek, Y., Bremler-barr, A., Koral, Y.: Efficient processing of multi-connection compressed web traffic, vol. 6640, pp. 52–65 (2011). https://doi.org/10.1007/978-3-642-20757-0_5

[14] Gailly, J.-l., Adler, M.: zlib compression library (2004)

[15] Adler, M.: "zlib compressed data format specification version 3.3". Technical report, RFC 1950 (May 1996). https://www.ietf.org/rfc/rfc1950.txt

[16] Elias, P.: Minimax optimal universal codeword sets. IEEE Transactions on Information Theory **29**(4), 491–502 (1983). https://doi.org/10.1109/TIT.1983.1056717

[17] Norris, J.R.: Markov Chains. Cambridge University Press, Cambridge (1997)

[18] Leavline, E.J., Singh, D.A.A.G.: Hardware implementation of lzma data

compression algorithm. International Journal of Applied Information Systems **5**, 52–56 (2013)

[19] Lindgren, G.: Shape and duration of clicks in modulated fm transmission. IEEE Transactions on Information Theory **30**(5), 728–735 (1984). https://doi.org/10.1109/TIT.1984.1056954

[20] Collin, L., Pavlov, I.: "lzma sdk (software development kit) 9.22 beta: Description of lzma compression". Technical report, Igor Pavlov (2015). https://www.7-zip.org/sdk.html

[21] Seward, J.: bzip2 and libbzip2 (1996)

[22] Burrows, M., Wheeler, D.J.: "a block-sorting lossless data compression algorithm". Technical report, Digital Equipment Corporation (1994)

[23] Salomon, D.: "Data Compression: The Complete Reference". Springer, Northridge (2004)

[24] Abedi, M., Malekpour, A., Luksch, P., Mojtabaei, M.: A method for compression of short unicode strings. (2017). 3rd International Conference on Computer Science Networks and Information Technology on 26th - 27th August 2017, University De Québec, Montreal, Canada

[25] Antirez, S.: "smaz - a short string compression library". In: "Proceedings of the 3rd Annual Redis Conference" (2012). https://antirez.com/misc/smv6.pdf

[26] Mouronte-López, M.L.: Modeling the public transport networks: A study of their efficiency. Complexity **2021**, 1–19 (2021). https://doi.org/10.1155/2021/3280777

[27] Mouronte-López, M.L., Gómez, J.: Exploring the mobility in the madrid community. Scientific Reports **13**, 904 (2023). https://doi.org/10.1038/s41598-023-27979-5

[28] Cheng, H.-P., Cheng, C.-S.: A support vector machine for recognizing control chart patterns in multivariate processes. (2007)

[29] Blanco, V., Puerto, J., Rodríguez-Chía, A.: On ℓ_p-support vector machines and multidimensional kernels (2017)

[30] Cutler, A., Cutler, D., Stevens, J.: Random Forests, vol. 45, pp. 157–176 (2011). https://doi.org/10.1007/978-1-4419-9326-7_5

[31] Rousseeuw, P.J.: Silhouettes: A graphical aid to the interpretation and validation of cluster analysis. Journal of Computational and Applied Mathematics **20**, 53–65 (1987). https://doi.org/10.1016/0377-0427(87)

90125-7

[32] Řezanková, H.: Different approaches to the silhouette cefficient calculation in cluster evaluation. (2018). 21st International Scientific Conference AMSE Applications of Mathematics and Statistics in Economics 2018

[33] Charrad, M., Ghazzali, N., Boiteau, V., Niknafs, A.: Nbclust: An r package for determining the relevant number of clusters in a data set. Journal of Statistical Software **61**, 1–36 (2014). https://doi.org/10.18637/jss.v061.i06

[34] Deutsch, L.P.: GZIP file format specification version 4.3. RFC Editor (1996). https://doi.org/10.17487/RFC1952. https://www.rfc-editor.org/info/rfc1952

[35] Seward, J.: bzip2 (1996). https://www.sourceware.org/bzip2/docs.html

[36] doc.rs: Smaz documentation. https://docs.rs/smaz/latest/smaz
